# Plastic-Degrading Microorganisms: Biodegradation Pathways and Habitat Origins

**DOI:** 10.3390/molecules31101638

**Published:** 2026-05-13

**Authors:** Martyna Jowita Żarska, Marcin Damian Jasiak, Patryk Mierzejewski, Klaudiusz Tomczyk, Jakub Małecki, Roksana Gudz, Aneta Krystyna Urbanek, Katarzyna Ewa Kosiorowska, Julia Alicja Dybka

**Affiliations:** 1 BIOSUS Student’s Scientific Club, Laboratory for Biosustainability, Institute of Biology, Wrocław University of Environmental and Life Sciences, Kożuchowska 5b, 51-631 Wrocław, Poland; 121730@student.upwr.edu.pl (M.J.Ż.); 123231@student.upwr.edu.pl (M.D.J.); 122625@student.upwr.edu.pl (P.M.); klaudiusz.tomczyk@upwr.edu.pl (K.T.); 125827@student.upwr.edu.pl (J.M.); 127381@student.upwr.edu.pl (R.G.); 2 Laboratory for Biosustainability, Institute of Biology, Wrocław University of Environmental and Life Sciences, Kożuchowska 5b, 51-631 Wrocław, Poland; aneta.urbanek@upwr.edu.pl; 3 Department of Applied Bioeconomy, Wrocław University of Environmental and Life Sciences, 37a, Chełmońskiego Str., 51-630 Wrocław, Poland; katarzyna.kosiorowska@upwr.edu.pl

**Keywords:** plastic biodegradation, polymer-degrading microorganisms, plastisphere, compost microbiota, soil microbiota, enzymatic degradation, PETase, polyolefins, polyesters, microbial consortia

## Abstract

Microbial biodegradation represents a promising approach to addressing global plastic pollution, yet the metabolic pathways and environmental origins of polymer-degrading microorganisms remain incompletely characterized. This review synthesizes current knowledge on biodegradation mechanisms across major polymer classes and identifies key environmental reservoirs harboring native plastic-degrading microbiota. Biodegradation pathways differ fundamentally according to polymer chemistry. Polyesters such as PET undergo hydrolytic cleavage by PETases and MHETases, releasing terephthalic acid and ethylene glycol for assimilation via the β-ketoadipate pathway and the TCA cycle. Biodegradable polyesters (PLA, PBAT, PHAs, PCL) are similarly hydrolyzed by cutinases, lipases, and depolymerases. In contrast, polyolefins (PE, PP) and polystyrene lack hydrolyzable bonds and require oxidative attack by laccases, peroxidases, and alkane monooxygenases, followed by β-oxidation to acetyl-CoA. Three principal environmental reservoirs supply plastic-degrading microorganisms: contaminated ecosystems including landfills and the plastisphere; soil microbiota contributing ligninolytic fungi and actinomycetes; and compost environments yielding thermostable enzymes such as leaf-branch compost cutinase. Across all environments, microbial consortia demonstrate superior degradation efficiency compared to single-species cultures, reflecting the enzymatic complexity required for complete polymer mineralization. Understanding these pathways and their environmental origins provides a foundation for biological plastic waste management strategies.

## 1. Introduction

Global plastic production has reached approximately 400 million tonnes per year, with nearly 40% of all manufactured plastics being discarded within a single year of production [1]. Due to their inherent resistance to biological degradation, synthetic polymers persist in the environment for decades to centuries, resulting in widespread and long-lasting contamination of both terrestrial and aquatic ecosystems [2,3]. The fragmentation of plastic debris into microplastics further exacerbates the problem by altering the physicochemical properties of soil, adversely affecting plant growth and animal health, and disrupting the composition and function of microbial communities [4]. Beyond ecological impacts, growing evidence indicates that plastic pollution poses a direct risk to human health. Micro- and nanoplastics have been detected in human blood, placenta, and brain tissue, and recent studies demonstrate that nanoplastics can cross the blood-brain barrier and accumulate in the central nervous system, where they are associated with neuroinflammation and behavioral alterations [5,6,7,8]. These findings underscore that plastic contamination is no longer solely an environmental concern but an emerging public health issue, further strengthening the urgency of developing efficient biological strategies for plastic removal and valorization. Conventional waste management strategies, including mechanical and chemical recycling as well as incineration, fail to fully address plastic leakage into the environment and do not enable the recovery of polymer-bound carbon within a circular economy framework [1,9]. In light of the escalating scale of plastic pollution, biological approaches to polymer degradation and upcycling are increasingly recognized as a promising approach for tackling this global environmental challenge.

Microorganisms belonging to various taxonomic groups have demonstrated the ability to interact with synthetic polymers, and some of them are capable of depolymerizing them. Bacteria are the dominant group among the described plastic-degrading isolates, accounting for approximately 56% of all reported cases of polyethylene terephthalate (PET) degradation, while fungi constitute a significant minority, accounting for approximately 32% [10,11]. Among bacteria, species of the genera *Pseudomonas*, *Alcanivorax*, and various *Actinobacteria* produce esterases, cutinases, and related hydrolases that act on polymers containing ester bonds, such as PET and selected aliphatic polyesters [12,13]. Filamentous fungi, including *Aspergillus* species, secrete oxidative and hydrolytic enzymes that cause measurable polymer weight loss under laboratory conditions [10,14]. In addition, microalgae, protists, and invertebrate gut microbiomes contribute to fragmentation, surface conditioning, and, in some systems, enzymatic depolymerization [10,11]. Main enzyme classes include PETases/cutins, MHETases, and general esterases, with dual enzyme systems (PETase + MHETase) having been functionally characterized for PET depolymerization [15,16]. This considerable diversity of microorganisms represents a valuable genetic and enzymatic resource for the development of biotechnological methods for plastic degradation.

In recent years, significant progress has been made in genetic and protein engineering aimed at increasing the catalytic activity, thermostability, and substrate range of enzymes that degrade plastics. Protein engineering approaches, including targeted mutagenesis and chimeric protein design, have led to the development of PETase/MHETase variants with increased activity towards PET and its intermediates, such as MHET [10]. Particular emphasis has been placed on improving enzymatic stability at industrially relevant temperatures and increasing activity towards semi-crystalline forms of PET [15]. Metabolic engineering of whole cells and consortia of microorganisms aims to create systems capable not only of depolymerizing plastics, but also of converting the resulting monomers into value-added products through bio-upcycling [1,9]. Strategies involving surface exposure and biofilm formation aim to maximize contact between modified cells, secreted enzymes, and the plastic surface [17]. Omics-based approaches, including metagenomics, enrichment cultures, and macrotranscriptomics, continue to reveal new potential hydrolases that serve as starting points for further modification [10]. Despite these remarkable technical achievements, the practical application of genetically modified strains faces significant obstacles.

**Figure 1:**
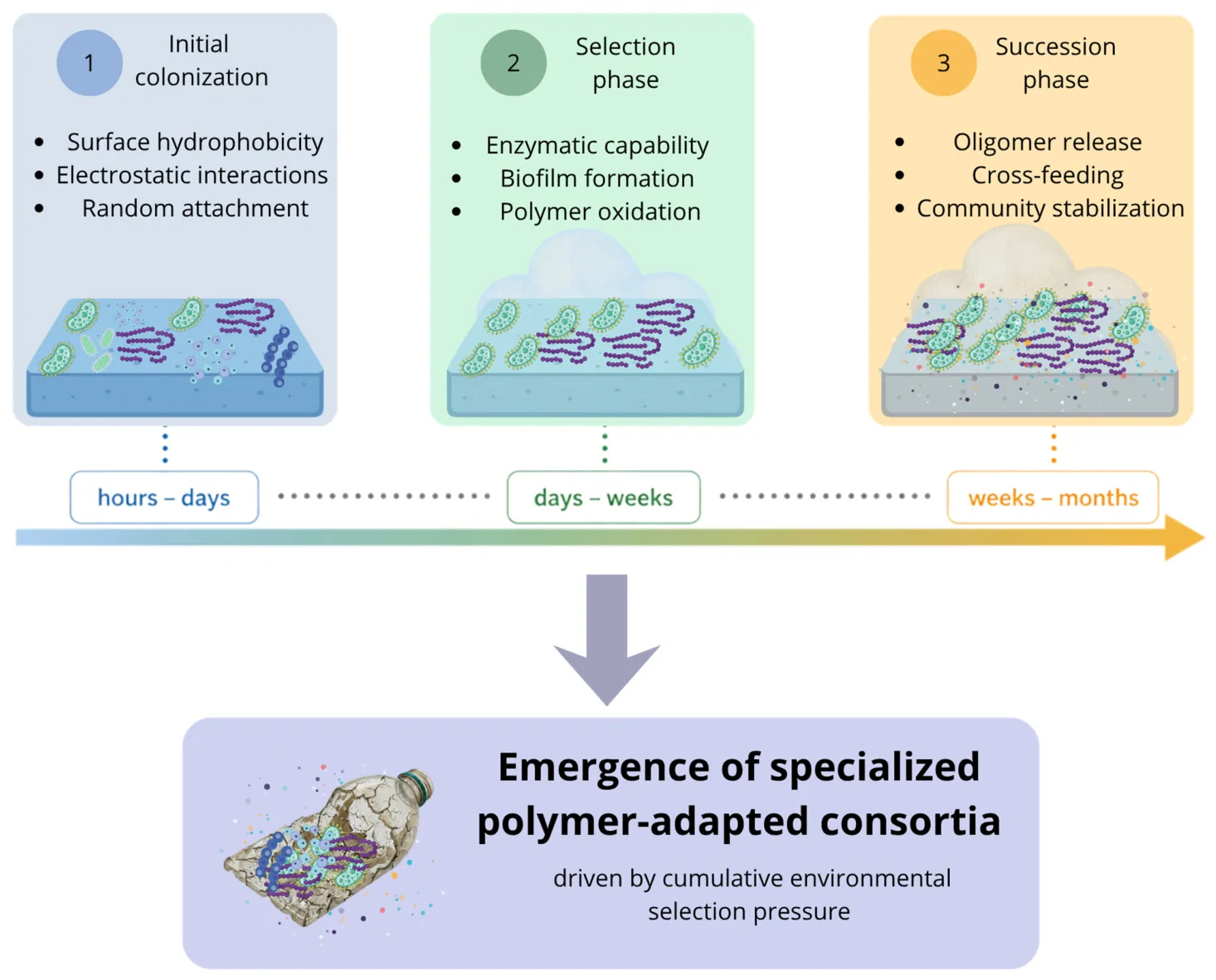
Selection-driven microbial succession on the plastic surface, illustrating the progressive replacement of pioneer colonizers by specialized polymer-degrading taxa under the selective pressure of the polymer substrate.

On plastic surfaces in the environment, this microbial diversity organizes itself into a selection-driven succession, in which early colonizers conditioning the polymer surface are progressively replaced by specialized degraders capable of metabolizing the polymer and its breakdown products, as illustrated in Figure 1 [18,19,20].

Given the limitations associated with the use of genetically modified strains, the search for efficient microorganisms in the natural environment is currently the most viable route to obtaining isolates suitable for industrial-scale application. Environmental isolates and microbial consortia have provided some of the most promising candidates with measurable degradation parameters, highlighting the critical importance of studying natural microbial communities alongside engineering-based approaches [21,22]. Recent studies of soil enrichment from mangrove forests, deep-sea sediments, and contaminated landfills have discovered new taxa and mixed communities capable of growing on PET or releasing depolymerization products, illustrating the ecological resilience and interspecies interactions that support polymer turnover [2,23,24]. Environmental isolates and consortia often exhibit mutual nutrition, mixed enzyme repertoires, and stability in complex matrices-features beneficial for industrial processes or closed bioprocesses [25]. The most suitable candidates combine measurable depolymerization activity (monomer release), scalability potential (thermostability or activity at process-relevant temperatures), and the ability to integrate with upcycling pathways. The practical way forward is to prioritize: (i) environmental screening supported by standardized activity tests, (ii) the selection of robust consortia or enzymes for closed bioreactor projects, and (iii) the combination of depolymerization with valorization pathways to recover value and balance process costs.

This review aims to summarize the latest and most effective environmental isolates that show potential for industrial application in the biological recycling of various synthetic polymers, with particular attention to three key environmental reservoirs of plastic-degrading microorganisms, contaminated sites and the plastisphere, soil microbiota, and composting habitats, and to the chemical mechanisms by which their enzymes act on each major polymer class. In particular, the article focuses on microorganisms isolated from the natural environment recently that show measurable degradation activity against major types of plastics, including polyethylene terephthalate (PET), polyethylene (PE), polystyrene (PS), and other polymers. The latest reports on effective bacteria, fungi, and consortia of microorganisms are presented, along with their enzymatic mechanisms and potential for use in biotechnological processes. Emphasis is placed on isolates that combine high degradation activity with features relevant from a practical point of view, such as thermostability, growth under industrial conditions, and compatibility with upcycling pathways. Through a comprehensive synthesis of current research, this review provides an overview of the state of knowledge on environmental sources of plastic-degrading microorganisms and points to directions for future research and industrial applications in the field of biological plastic waste management.

## 2. Methodology

A systematic literature search was conducted using three major scientific databases: Scopus, Web of Science, and PubMed. The search strategy was built around five interconnected concept clusters. The first cluster covered biodegradation processes and enzymatic mechanisms, using terms such as “plastic degradation”, “polymer biodegradation”, “plastic depolymerization”, and “enzymatic degradation”. The second cluster encompassed the polymer types addressed in this review, including polyethylene (LDPE, HDPE), polypropylene, polystyrene, polyethylene terephthalate, polylactic acid, polyhydroxyalkanoates, polycaprolactone, polybutylene adipate terephthalate, polyurethane, and polybutylene succinate. The third cluster targeted the biological agents of degradation, comprising terms such as “microorganism”, “bacteria”, “fungi”, “microbial consortium”, “isolate”, “gut microbiome”, and “plastisphere”. The fourth cluster addressed the environmental sources and habitats of plastic-degrading microorganisms, including “soil”, “compost”, “composting”, “landfill”, “marine”, “wastewater”, “contaminated soil”, and “insect gut”. The fifth cluster, introduced during the revision process to comprehensively cover the contribution of culture-independent approaches to the discovery of plastic-degrading enzymes, targeted molecular and metagenomic methodologies. It included terms such as “metagenomics”, “metagenomic mining”, “functional metagenomics”, “shotgun metagenomics”, “metagenome-assembled genomes”, “MAGs”, “environmental DNA”, “eDNA”, “uncultured microorganisms”, “non-cultivable bacteria”, “hidden Markov models”, and “in silico enzyme discovery”. The five clusters were combined using the Boolean operator AND, while records focused exclusively on abiotic processes, photodegradation, or chemical recycling were excluded using the NOT operator. No publication date restrictions were applied, and the search was conducted up to March 2026. The included literature comprised peer-reviewed journal articles, book chapters, and scientific monographs, while non-peer-reviewed sources, conference abstracts, and duplicate records were excluded. In addition, the reference lists of all retrieved publications were manually screened to identify relevant studies not captured by the database search. This study is a narrative review; therefore, a formal PRISMA guidelines protocol was not applied. However, the literature selection process was designed to ensure transparency and relevance.

## 3. Biodegradation Mechanisms Across Polymer Types

The biodegradation of blended plastic waste remains a major challenge, mainly due to the variety of degradation mechanisms required for different types of polymers. Each class of polymers has a distinct chemical structure that determines its susceptibility to attack by microorganisms, requiring specific enzymatic mechanisms and, consequently, specialized microorganisms. Therefore, biodegradation pathways can be systematically classified according to polymer type, reflecting fundamental differences in chemical bonds, crystallinity, and hydrophobicity that determine accessibility to microorganisms.

### 3.1. Polyolefins

Polyethylene (PE) appears in two main types, differing in properties and applications. The first one—LDPE (low-density polyethylene)—is soft and flexible, thus widely used in the production of wrapping films, grocery bags, and shipping bags [26]. The second type, HDPE (high-density polyethylene), is commonly used to make bottles, canisters, water pipes, and storage containers. HDPE is the polymer most commonly found in soil, lake and sea sediments, and freshwater and seawater [27]. Another important representative of polyolefins is polypropylene (PP), widely used in the food industry for packaging products, as well as in the production of nonwovens-including disposable protective masks-and bottle closures. Polypropylene is highly resistant to chemicals, but at the same time it easily breaks down into microplastic particles, which means it remains in ecosystems for a long time [28].

#### 3.1.1. LDPE (Low-Density Polyethylene) and HDPE (High-Density Polyethylene)

Of all types of polyethylene, LDPE is the most susceptible to microbial degradation. This is a direct result of its structure: side branches limit the tight packing of chains, which translates into lower crystallinity and thus greater accessibility to bacterial enzymes [29]. Studies confirm that microorganisms primarily attack the amorphous regions of the polymer, which are more loosely packed and more accessible to degradative enzymes. As degradation progresses, the crystalline areas remain relatively intact, while the amorphous regions are gradually broken down [29].

HDPE is characterized by significantly higher resistance to microbial degradation than LDPE or LLDPE (Linear Low-Density Polyethylene). This is a direct result of its structure—the tight packing of linear polymer chains and high crystallinity effectively limit the access of bacterial enzymes to the interior of the material [30]. Since microorganisms primarily attack the amorphous regions of the polymer, and these are few and difficult to access in HDPE, the biodegradation process is much slower. In addition, the higher molecular weight of HDPE makes it difficult to cut the chains enzymatically. Among the polyethylene types studied, HDPE exhibits the lowest susceptibility to thermo-mechanical degradation under repeated extrusion conditions (LDPE > LLDPE > HDPE), although this ranking is of a general nature, Felgel-Farnholz et al. (2023) demonstrated that different types of PE degrade via distinct mechanisms (chain scission dominates in HDPE and LLDPE, whereas cross-linking initially predominates in LDPE), which makes it difficult to unambiguously rank them on a single sensitivity scale [31].

**Figure 2:**
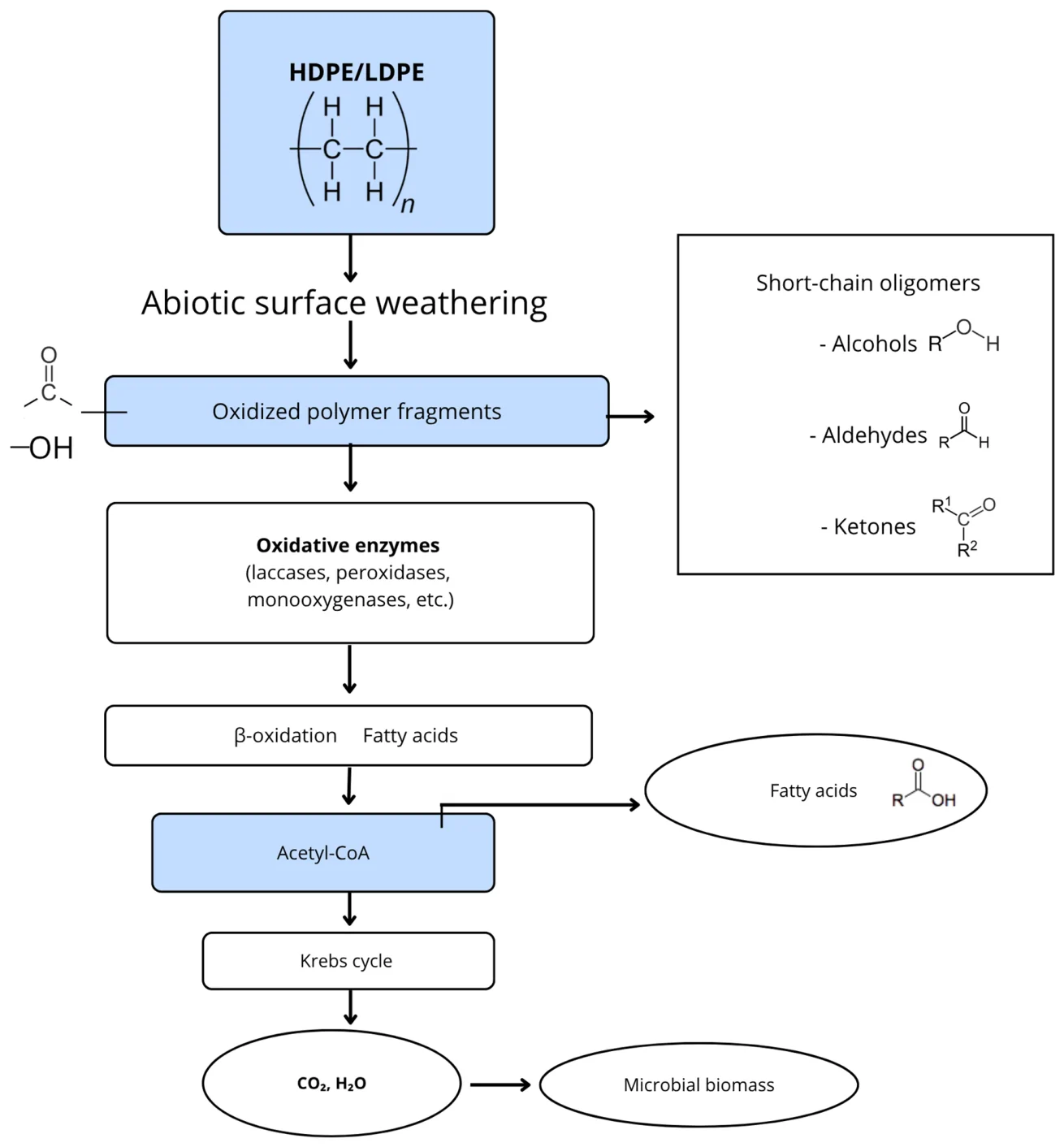
Schematic of the polyethylene degradation metabolic pathway. Scheme prepared by the authors based on KEGG metabolic pathway analysis (Kyoto Encyclopedia of Genes and Genomes), specifically referring to the reference maps map00071 (Fatty acid degradation), map00620 (Pyruvate metabolism), and map00020 (Citrate cycle/TCA cycle) for the β-oxidation of polyethylene-derived fatty acids and acetyl-CoA mineralization, and complemented with the literature data on alkane monooxygenase (alkB), cytochrome P450, laccase, and peroxidase activity cited in the corresponding section of this manuscript.

The biodegradation process of both LDPE and HDPE occurs in several stages and it is shown in Figure 2. Initially, abiotic weathering of the polymer surface occurs, leading to the formation of oxygen-containing functional groups (carbonyl and hydroxyl), thus increasing the susceptibility of the polymer to enzymatic attack [32]. Subsequently, extracellular oxidizing enzymes: multicopper oxidases, laccases, peroxidases, GPx, cytochrome P450 monooxygenases, and alkane monooxygenases, which introduce oxygen atoms and cause chain cleavage, yielding alcohols, aldehydes, ketones, and short oxidized oligomers [31,32,33]. The oxidized fragments are then converted into fatty acids, such as palmitic acid detected in bacterial cultures, as well as short-chain alkanes and alcohols [32].

The fatty acids formed as a result of depolymerization undergo β-oxidation to acetyl-CoA, which then enters the tricarboxylic acid cycle (TCA cycle, Krebs cycle) for energy production and cell biomass synthesis [30,34,35]. Intermediate metabolites identified during LDPE degradation include alcohols, aldehydes, ketones, mono- and dicarboxylic acids, and medium-chain fatty acids, including palmitic acid [32,33]. The end products of complete mineralization are CO_2_, H_2_O, and microbial biomass, resulting from the oxidation of acetyl-CoA in the TCA cycle; however, complete mineralization is only observed under conditions of prolonged incubation or when microbial consortia are used [32,35]. It should be emphasized that throughout this review, the term “complete mineralization” refers to the stoichiometric end-products of full substrate oxidation through the TCA cycle (CO_2_, H_2_O, and microbial biomass), as inferred from KEGG metabolic pathway analysis. Experimental confirmation of mineralization in the cited primary literature is based on respirometric assays measuring CO_2_ evolution (Sturm test, ISO 14855, ASTM D5338) and on isotope-tracing experiments using ^13^C- or ^14^C-labeled substrates, which together constitute the methodological gold standard for distinguishing genuine mineralization from polymer fragmentation, surface erosion, or partial assimilation into microbial biomass.

#### 3.1.2. PP (Polypropylene)

Polypropylene is a plastic with moderate to low susceptibility to microbial degradation. The presence of methyl groups in the main chain creates steric hindrance that limits enzymatic access to the chemical bonds in the polymer backbone As with other polyolefins, PP does not have easily hydrolyzed bonds in its structure, such as the ester bonds present in PET or PLA. The degradation of PP therefore requires the action of oxidative enzymes, which are less common and act more slowly. The high hydrophobicity of the PP surface further hinders colonization by microorganisms.

**Figure 3:**
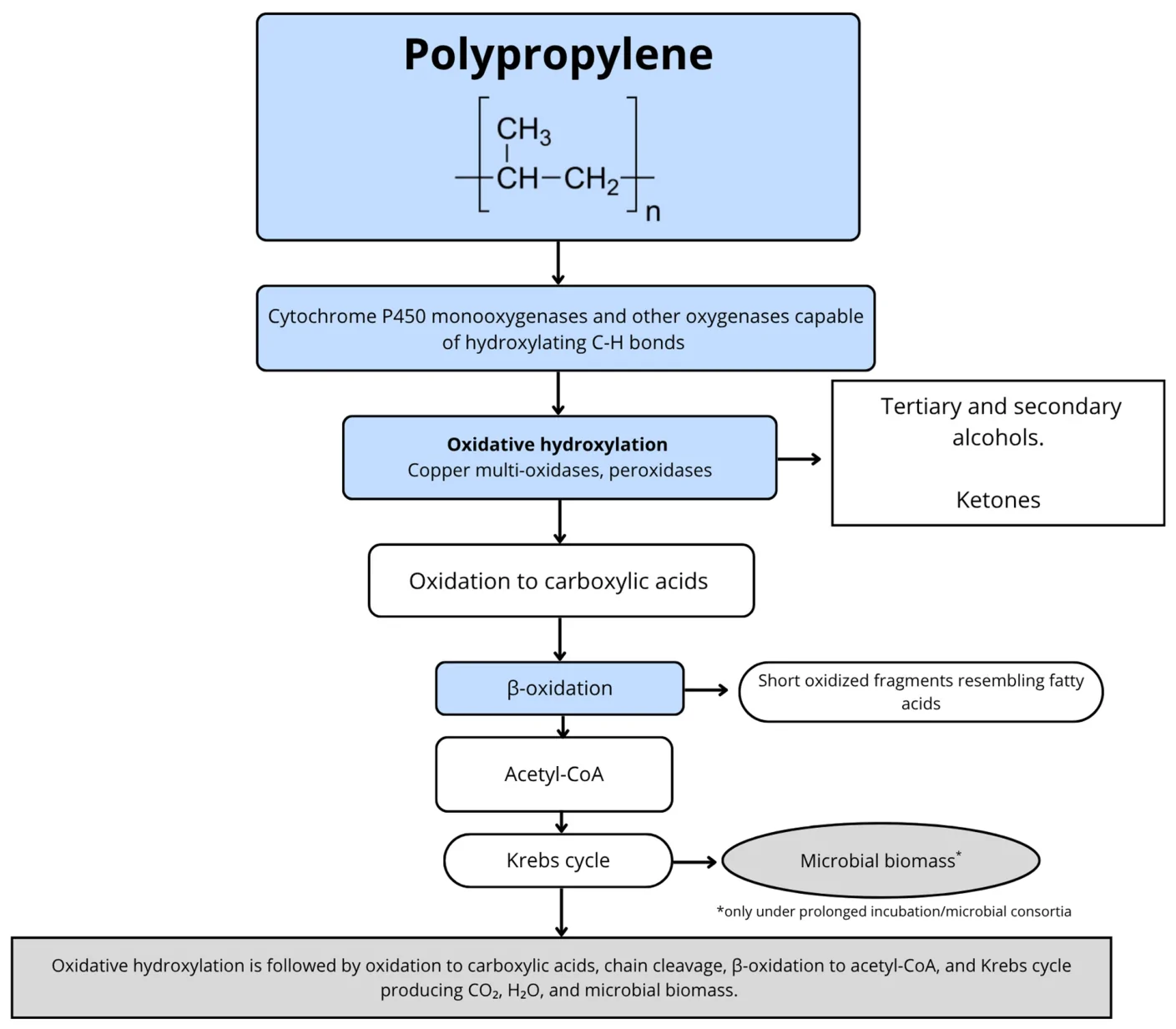
Enzymatic oxidation and β-oxidation route in polypropylene biodegradation. Scheme prepared by the authors based on KEGG metabolic pathway analysis (Kyoto Encyclopedia of Genes and Genomes), specifically referring to the reference maps map00071 (Fatty acid degradation), map00640 (Propanoate metabolism), and map00020 (Citrate cycle/TCA cycle) for the oxidation and β-oxidation of polypropylene-derived hydrocarbon fragments, and complemented with the literature data on alkB-encoded alkane monooxygenase activity and biofilm-mediated polymer colonization cited in the corresponding section of this manuscript.

The biodegradation of polypropylene follows a mechanism similar to that of polyethylene (Figure 3), but with significant differences. The main role is played by enzymes from the alkane monooxygenase group, encoded, among others, by the *alkB* gene. The process begins with the oxidation of polymer chains and the introduction of oxygen-containing functional groups. An interesting discovery is the possibility of degrading PP in liquefied form, which suggests that preliminary “loosening” of the polymer structure significantly facilitates enzymatic attack. The formation of a dense biofilm on the surface of PP is a major factor determining the effectiveness of degradation.

### 3.2. PS (Polystyrene)

Polystyrene (PS) is an aromatic polymer composed of repeating styrene units containing phenyl side groups, which provide high chemical stability and make PS one of the most difficult plastics to biodegrade. Nevertheless, an increasing number of microorganisms and enzymes capable of degrading PS have been identified, with the intestinal microflora of insects playing a particularly important role [22,36,37]. The main enzymes initiating PS biodegradation are oxidases and peroxidases with high redox potential, including laccases, decolorizing peroxidases (DyP) and manganese peroxidases (MnP), which catalyze the oxidative depolymerization of the polymer surface, increasing its hydrophilicity and releasing oligomers and monomers [36,38,39]. Serine hydrolases and other hydrolytic enzymes further cleave the oxidized bonds in *Pseudomonas* and other intestinal isolates [40]. Additionally, alkane monooxygenases transcriptionally induced in some *Pseudomonas* strains after exposure to PS, oxidize alkyl chains and side groups on partially degraded PS fragments [38].

After the release of the styrene monomer, its catabolism proceeds mainly via the side-chain oxidation pathway, a canonical bacterial pathway involving three consecutive enzymes: styrene monooxygenase (SMO), a two-component FAD-dependent monooxygenase that epoxidizes styrene to styrene oxide; styrene oxide isomerase (SOI), a membrane-bound enzyme that converts styrene oxide to phenylacetaldehyde; and phenylacetaldehyde dehydrogenase (PAD), an NAD^+^-dependent enzyme that oxidizes phenylacetaldehyde to phenylacetic acid (PAA) [41]. PAA is then activated to phenylacetyl-CoA and directed to the phenylacetyl-CoA catabolon, where hydroxylation and ring cleavage generate central metabolites for energy production and biomass synthesis [36,41]. Experimental detection of styrene oxide and phenylacetic aldehyde during fungal PS processing confirmed the presence of these intermediate products in environmental systems [42]. An alternative pathway involves direct cleavage of the aromatic ring by ring-cleaving dioxygenases, which dearomatize the phenyl group of styrene or its oxidized derivatives to produce dihydrodiols and open-chain carboxylates. Evidence for this pathway, including FTIR-confirmed dearomatization and a strict requirement for molecular oxygen, has been reported for *Exiguobacterium* sp. RIT594 [43]. Recent transcriptomic and heterologous expression studies have also identified kynurenine monooxygenase (Kmo) and 4-hydroxyphenylpyruvate dioxygenase (Hpd) from *Stenotrophomonas maltophilia* ZSL2493, an isolate from the gut of *Tenebrio molitor* larvae, as the key depolymerizing enzymes responsible for direct cleavage of PS polymer chains. The parent strain achieved a polystyrene biodegradation efficiency of 14.7% within 30 days of incubation, whereas heterologous expression of the two enzymes in recombinant hosts resulted in measurable release of styrene monomer, with yields of 1.37 μg mL^−1^ for the Kmo-treated group and 0.95 μg mL^−1^ for the Hpd-treated group after 30 days, providing direct evidence of enzymatic depolymerization of the polymer backbone [41].

**Figure 4:**
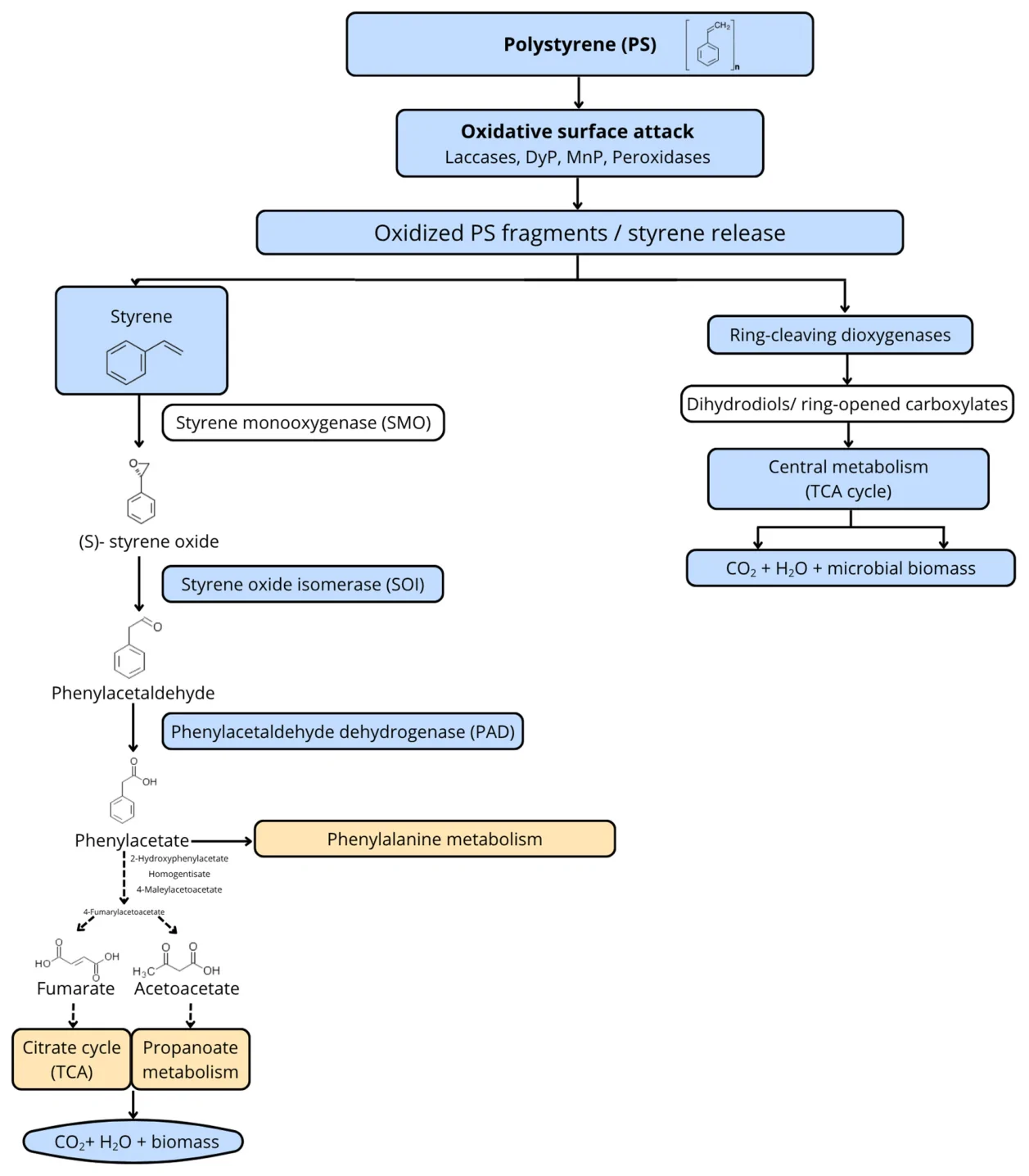
Mechanistic routes of polystyrene depolymerization and styrene assimilation. Scheme prepared by the authors based on KEGG metabolic pathway analysis (Kyoto Encyclopedia of Genes and Genomes), specifically referring to the reference maps map00643 (Styrene degradation) for the canonical SMO-SOI-PAD pathway converting styrene to phenylacetic acid (PAA), map00360 (Phenylalanine metabolism) for the phenylacetyl-CoA catabolon and ring-cleavage steps, map00362 (Benzoate degradation) for the β-ketoadipate pathway, and map00020 (Citrate cycle/TCA cycle) for terminal mineralization. The scheme was complemented with the literature data on oxidative depolymerization by laccases, DyP and MnP peroxidases, alkane monooxygenases, and ring-cleaving dioxygenases cited in the corresponding section of this manuscript.

Mechanistic routes of polystyrene depolymerization and styrene assimilation is shown in Figure 4.

A wide range of bacteria and fungi are involved in PS degradation. Bacteria of the genus *Pseudomonas* (including *P. putida* and *P. aeruginosa*) have been repeatedly isolated from environments contaminated with plastics and from the intestines of insects, showing chemical changes on the surface and induction of alkane monooxygenase and alcohol dehydrogenase during PS incubation [40]. *Exiguobacterium* spp. forms biofilms on PS surfaces and produces indentations associated with reduced hydrophobicity and the appearance of carboxyl and hydroxyl groups [43,44]. *Stenotrophomonas* spp. from insect guts encodes both the canonical styrene degradation pathway and potential depolymerizers (Kmo, Hpd) associated with PS depolymerization and styrene release [41]. *Microbacterium esteraromaticum* SW3 uses PS as its sole carbon source, and manganese peroxidase and lipase activity influences polymer conversion [38]. Among fungi, white rot species such as *Pleurotus ostreatus* and *Phanerochaete chrysosporium* showed PS weight loss, FTIR evidence of new carbonyl and hydroxyl groups, and CO_2_ release in Sturm tests [42,45].

The gut microbiota of insects is a particularly effective PS biodegradation system. The gut communities of the yellow mealworm (*Tenebrio molitor*), superworm (*Zophobas atratus*), and greater wax moth (*Galleria mellonella*) contain PS-degrading bacteria and are essential for the rapid depolymerization and mineralization observed in vivo; inhibition of gut bacteria in mealworms with antibiotics resulted in the abolition of both PS depolymerization and mineralization [40,44,46,47]. Experiments using ^13^C-labeled polystyrene confirmed the incorporation of polymer-derived carbon into microbial biomass and its conversion to ^13^CO_2_, providing direct isotopic evidence of complete mineralization rather than mere fragmentation; this carbon flux was attributed to the metabolic activity of gut bacteria, since antibiotic suppression of the gut microbiota abolished both PS depolymerization and CO_2_ release [44].

Intermediate metabolites identified during PS biodegradation include styrene, styrene oxide, phenylacetaldehyde, phenylacetic acid, 2-phenylethanol, 1-phenyl-1,2-ethanediol, alkylbenzenes (*p*-xylene, ethylbenzene), substituted benzoates, and open-chain carboxylates [37,42,43]. The end products of complete mineralization are CO_2_, H_2_O, and microbial biomass; however, mineralization validated by direct CO_2_-evolution measurements (Sturm-type respirometry) or ^13^C-tracer assays has been most consistently demonstrated in insect gut systems and in selected white-rot fungal cultures, whereas isolated environmental bacterial strains more commonly achieve only partial depolymerization and accumulation of oxidized products without confirmed terminal oxidation [36,42,44].

### 3.3. PET (Polyethylene Terephthalate)

**Figure 5:**
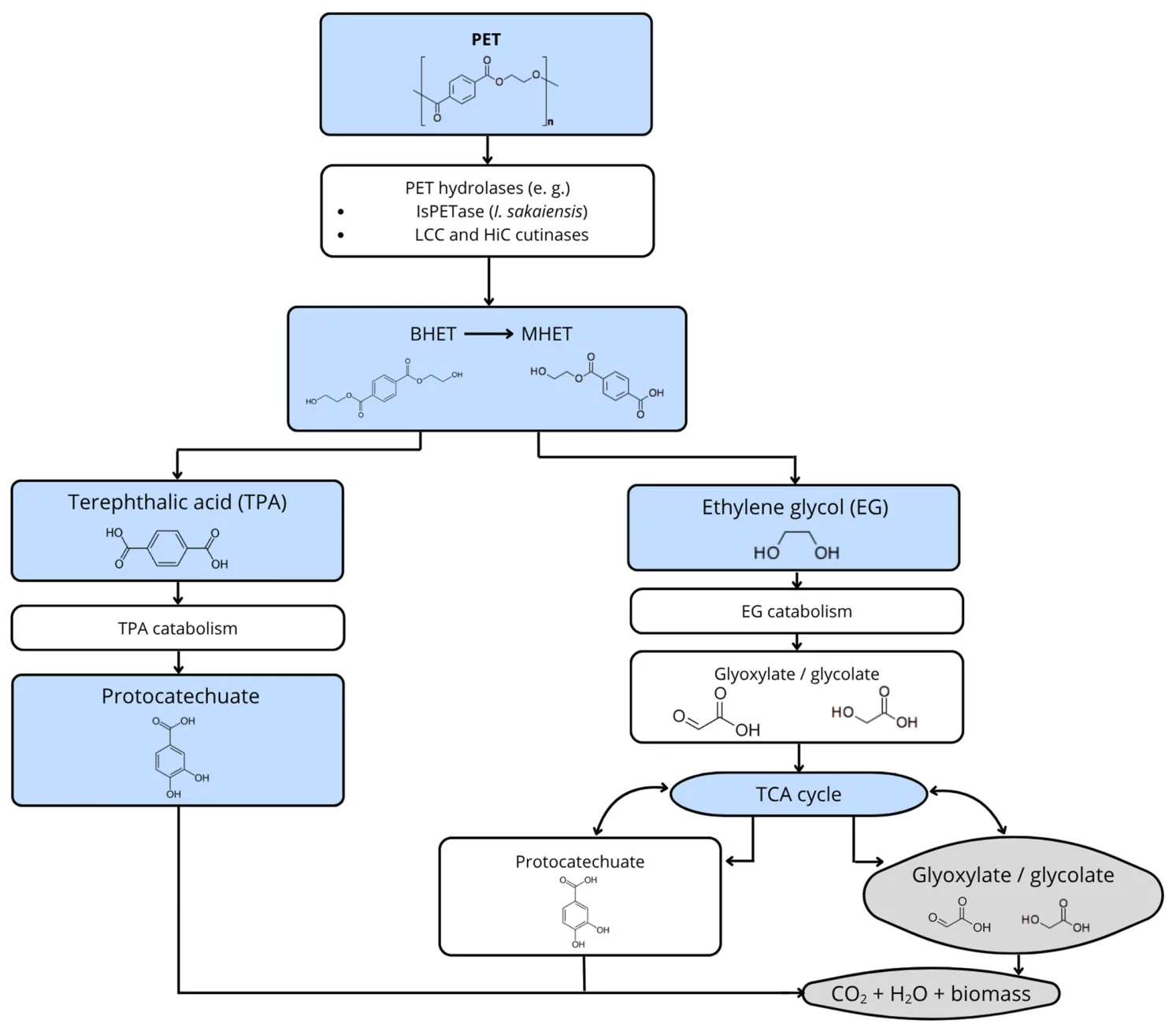
Enzymatic hydrolysis and metabolic assimilation during PET biodegradation. Scheme prepared by the authors based on KEGG metabolic pathway analysis (Kyoto Encyclopedia of Genes and Genomes), specifically referring to the reference maps map00624 (Polycyclic aromatic hydrocarbon degradation, including module M00624—Terephthalate degradation, terephthalate ⇒ 3,4-dihydroxybenzoate) for TPA conversion to protocatechuate via TphAabc and TphB, map00362 (Benzoate degradation) for the protocatechuate branch of the β-ketoadipate pathway leading to acetyl-CoA and succinyl-CoA, map00630 (Glyoxylate and dicarboxylate metabolism) for ethylene glycol oxidation to glycolaldehyde, glycolate, and glyoxylate, and map00020 (Citrate cycle/TCA cycle) for terminal mineralization. The scheme was complemented with the literature data on extracellular PET hydrolases (IsPETase, LCC, HiC) and MHETase activity cited in the corresponding section of this manuscript.

Polyethylene terephthalate (PET) is a polyester polymer widely used in the packaging industry. Unlike polyolefins, PET is biodegradable through ester bond hydrolysis, which makes it more susceptible to enzymatic attack by microorganisms. The PET biodegradation process has been thoroughly characterized, and several bacterial strains, as well as genetically modified host organisms, have demonstrated the ability to depolymerize PET and subsequently metabolize its monomers [48,49,50]. Enzymatic hydrolysis and metabolic assimilation during PET biodegradation are shown in Figure 5.

The main enzymes involved in PET biodegradation are extracellular PET hydrolases including IsPETase from *Ideonella sakaiensis*; leaf-branch compost cutinase (LCC), a thermostable α/β-hydrolase originally identified by Sulaiman et al. through functional metagenomic screening of a fosmid library prepared from leaf-branch compost, with a sequence most closely related to cutinases of thermophilic actinomycetes such as *Thermobifida fusca* (57.4% identity) and *Thermomonospora curvata* (59.7% identity), which strongly suggests an uncultured thermophilic actinobacterium as the source organism; cutinase from the thermophilic fungus *Humicola insolens* (HiC); cutinase from *Humicola insolens* (HiC); and other PETases and cutinases that catalyze the hydrolysis of ester bonds in the PET backbone. These enzymes convert PET into oligomeric and monomeric intermediates, mainly bis(2-hydroxyethyl) terephthalate (BHET) and mono(2-hydroxyethyl) terephthalate (MHET) [48,51,52]. MHET is then hydrolyzed by MHETase or related esterases, resulting in the formation of terephthalic acid (TPA) and ethylene glycol (EG) [53]. At the intracellular level, enzymes of the aromatic catabolic pathway, including TPA dioxygenases and products of the *tph* operon genes, convert TPA to protocatechuic acid (PCA), which is further processed by ring-opening enzymes that direct carbon into central metabolism [53,54].

Microorganisms capable of PET degradation include a number of environmental isolates and consortia. Bacteria of the genera *Pseudomonas* and *Bacillus* have been shown to colonize PET surfaces and utilize BHET as a carbon source. In addition, genetically modified strains of *Pseudomonas putida* expressing heterologous PET hydrolases and TPA catabolic genes have been developed to increase degradation efficiency [10,55].

The metabolic pathway for PET biodegradation proceeds through several well-defined steps. Initially, PET hydrolases (e.g., IsPETase, LCC, or HiC) cleave the ester bonds in the polymer chains, producing oligomers as well as BHET and MHET monomers. Next, MHETase and associated esterases convert MHET and BHET residues into TPA and EG [10].

TPA catabolism involves its uptake and conversion by TPA dioxygenase and *tph* operon genes into protocatechuic acid (PCA). PCA is then subjected to aromatic ring cleavage via the β-ketoadipate pathway or related pathways, resulting in the formation of acetyl-CoA and succinyl-CoA, which enter the tricarboxylic acid (TCA) cycle [53,54]. EG catabolism proceeds via oxidation by alcohol and aldehyde dehydrogenases to glycolaldehyde and then to glycoxylate/glycolate intermediates, which enter central metabolism via the glycoxylate or glycerate pathways, ultimately generating acetyl-CoA for the TCA cycle. Intermediate metabolites identified during enzymatic and microbial degradation of PET include BHET, MHET, TPA, EG, protocatechuate, and intermediate products of the glycoxylate/glycolate pathway. The end products of complete mineralization are CO_2_, H_2_O, and microbial biomass, resulting from the conversion of TPA and EG through aromatic ring cleavage and C_2_ catabolism to acetyl-CoA, followed by oxidation in the TCA cycle [48,56].

Genetically modified whole-cell systems have integrated PET hydrolase expression with monomer assimilation pathways, including the *tph* operon for TPA catabolism and evolved pathways for EG and 1,4-butanediol utilization. These systems have demonstrated a complete cascade from hydrolysis to assimilation in laboratory strains, representing a promising route for the biological upcycling of PET waste [57].

### 3.4. Common Biodegradable Plastics

**Figure 6:**
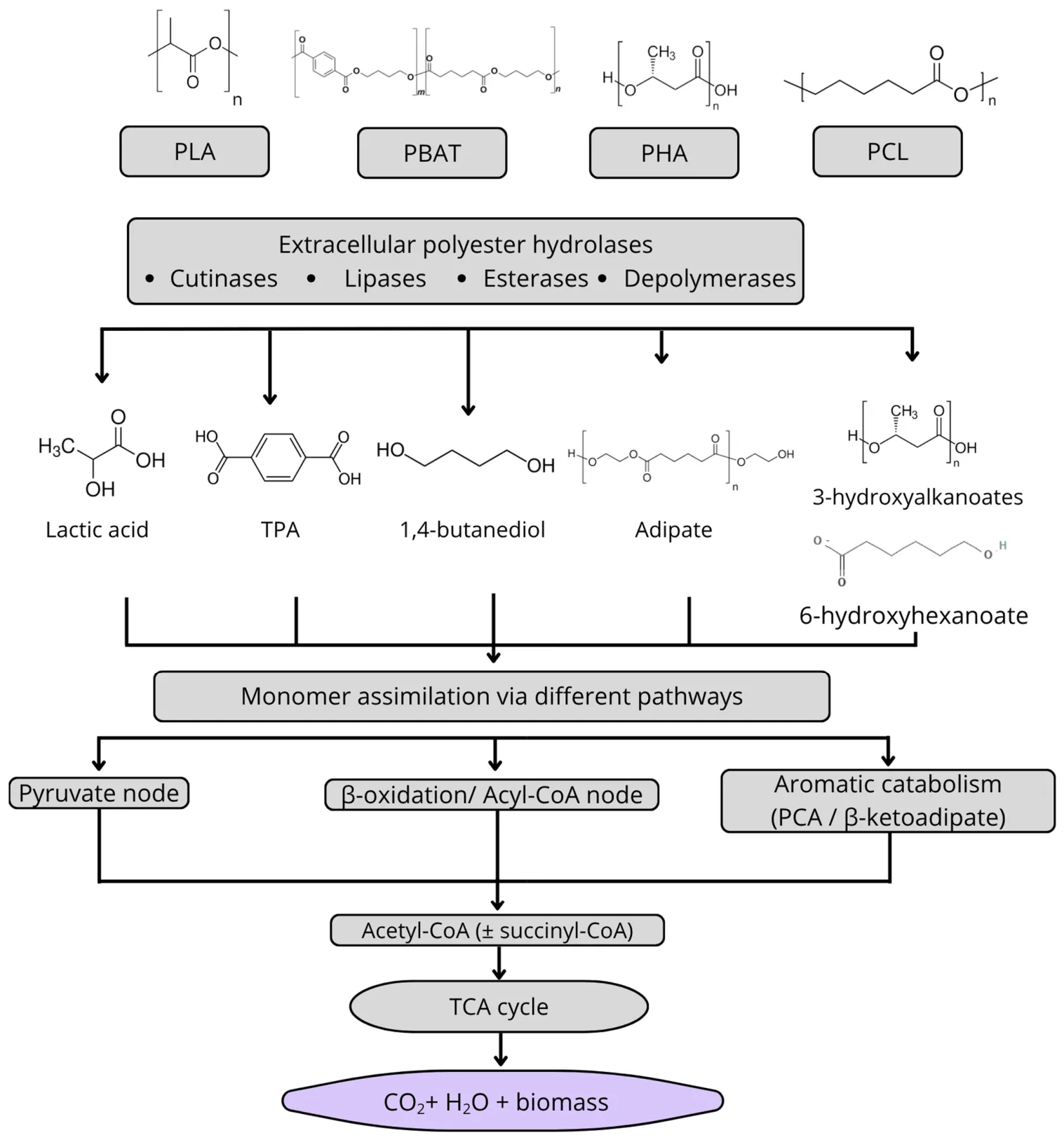
Common metabolic pathways in biodegradable polyesters degradation. Scheme prepared by the authors based on KEGG metabolic pathway analysis (Kyoto Encyclopedia of Genes and Genomes), specifically referring to the reference maps map00620 (Pyruvate metabolism) for the conversion of L-/D-lactate to pyruvate and acetyl-CoA in the PLA pathway, map00624 (Polycyclic aromatic hydrocarbon degradation, including module M00624—Terephthalate degradation) and map00362 (Benzoate degradation, β-ketoadipate pathway) for the aromatic branch of PBAT degradation, map00650 (Butanoate metabolism) for the PHA degradation route via 3-hydroxybutyrate, acetoacetate, and acetoacetyl-CoA, map00071 (Fatty acid degradation) for the β-oxidation of PCL-derived 6-hydroxyhexanoic acid and short-chain hydroxy acids, and map00020 (Citrate cycle/TCA cycle) for terminal mineralization. The scheme was complemented with the literature data on extracellular ester-bond hydrolysis by PLA depolymerases, cutinases (AaCut4, AaCut10), PHA depolymerases (ePhaZ/PhaZ), and lipases/esterases cited in the corresponding sections of this manuscript.

Although biodegradable plastics such as PLA, PBAT, PHAs, and PCL are designed to be more environmentally benign than their conventional counterparts, their rapidly increasing global production, driven by legislative pressure and consumer demand, means that substantial volumes of these materials are entering waste streams and natural environments. Understanding their biodegradation pathways is therefore essential to ensure that their end-of-life fate aligns with the environmental benefits they are expected to deliver. Common metabolic pathways in biodegradable polyesters degradation are shown in Figure 6.

#### 3.4.1. PLA (Polylactic Acid)

Polylactic acid (PLA) is a biodegradable polyester derived from renewable raw materials such as corn starch. Its biodegradation occurs mainly through the hydrolysis of ester bonds by specific microbial enzymes, resulting in the formation of lactic acid, which is easily assimilated by microorganisms [58].

The metabolic pathway for PLA degradation begins with the hydrolysis of extracellular ester bonds, in which PLA depolymerases, proteinase K, and lipases cleave the polymer into short oligomers and then lactic acid monomers. Lactic acid is then converted to pyruvate by lactate dehydrogenases and directed to central metabolism as acetyl-CoA, which enters the tricarboxylic acid (TCA) cycle. The main intermediate metabolites are short-chain PLA oligomers and lactic acid (L- and D-lactate); the stoichiometric end-products of full lactate oxidation through the TCA cycle are CO_2_, H_2_O, and microbial biomass, as confirmed by industrial composting respirometric standards (ISO 14855) for PLA [59,60].

#### 3.4.2. PBAT (Polybutylene Adipate Terephthalate)

Polybutylene adipate terephthalate (PBAT) is a biodegradable aliphatic-aromatic copolyester composed of butylene adipate (aliphatic) and butylene terephthalate (aromatic) segments. Its biodegradation proceeds primarily through ester bond hydrolysis catalyzed by cutinases and other polyester hydrolases, yielding terephthalic acid (TPA), 1,4-butanediol, and adipate-related fragments [61]. Cutinasas such as AaCut4 and AaCut10 from the marine fungus *Alternaria alternata* FB1 are particularly important enzymes, exhibiting high PBAT depolymerization efficiency at moderate temperatures, achieving near-quantitative TPA recovery, and enabling monomer recycling or further metabolic assimilation [62].

Under aerobic conditions, the metabolic pathway of PBAT degradation is initiated by extracellular cutinase-mediated ester hydrolysis, releasing TPA, 1,4-butanediol, and adipate oligomers and monomers [63]. TPA is subsequently catabolized via the *tph*/protocatechuate route, proceeding through protocatechuate and the β-ketoadipate pathway to acetyl-CoA. 1,4-Butanediol is oxidized and assimilated through C_4_/C_2_ metabolic routes; notably, laboratory evolution of *Pseudomonas putida* has demonstrated the acquisition of efficient 1,4-butanediol catabolism. The final products of aerobic mineralization are CO_2_, H_2_O, and microbial biomass [64].

Under anaerobic conditions, abiotic and microbial hydrolysis of PBAT generates oligomers and monomers that are further processed through acetogenesis to acetate, followed by methanogenesis, yielding CH_4_ and CO_2_ as the terminal products. The principal intermediate metabolites identified across both aerobic and anaerobic systems include TPA, 1,4-butanediol, adipate fragments, protocatechuate, and β-ketoadipate pathway intermediates [65].

#### 3.4.3. PHAs (Polyhydroxyalkanoates)

Polyhydroxyalkanoates (PHAs) are a family of biodegradable polyesters produced naturally by bacteria as intracellular granules that store carbon and energy. Due to their microbiological origin, the enzymology and metabolism of PHAs are much better characterized than those of synthetic polyesters. The main enzymes mediating PHA biodegradation are extracellular PHA depolymerases (ePhaZ family) and intracellular PHA depolymerases (PhaZ), which hydrolyze polymer chains into 3-hydroxyalkanoate monomers. For short-chain PHAs such as poly(3-hydroxybutyrate) (PHB), further processing involves 3-hydroxybutyrate dehydrogenase and acetoacetyl-CoA thiolase. Numerous soil and marine bacteria, including *Pseudomonas* and *Streptomyces* species, as well as many PHA-accumulating taxa and some fungi, express PHA depolymerizers and utilize PHA as their sole carbon source [66,67].

The metabolic pathway for PHA degradation begins with extracellular or intracellular depolymerization by ePhaZ/PhaZ, releasing 3-hydroxyalkanoate monomers (e.g., 3-hydroxybutyrate in the case of PHB). The monomer is then oxidized by 3-hydroxybutyrate dehydrogenase to acetoacetate, which is activated to acetoacetyl-CoA and cleaved by thiolase into two molecules of acetyl-CoA. Acetyl-CoA enters the tricarboxylic acid (TCA) cycle, where it is completely oxidized via the electron transport chain. Well-documented intermediates include 3-hydroxyalkanoates, acetoacetate, acetoacetyl-CoA, and short-chain forms of acyl-CoA; the stoichiometric end-products of acetyl-CoA oxidation through the TCA cycle are CO_2_, H_2_O, and microbial biomass. Importantly, the high specificity of PHA depolymerases supports rapid mineralization rates that have been quantitatively validated by CO_2_-evolution measurements in respirometric assays [68,69,70].

#### 3.4.4. PCL (Polycaprolactone)

Polycaprolactone (PCL) is a biodegradable aliphatic polyester widely used in biomedicine and packaging. Its biodegradation occurs mainly through hydrolysis of the ester bond catalyzed by extracellular lipases, esterases, and cutinases produced by a wide range of bacteria and fungi. Bacteria of the genera *Pseudomonas*, *Streptomyces*, and *Alcaligenes*, as well as fungi of the genera *Aspergillus* and *Fusarium*, exhibit the activity of these enzymes and show PCL degradation activity, with *Rhodococcus* strains exhibiting particularly strong hydrolytic activity and producing carboxylic acids detectable in the culture supernatant [21,71,72].

The metabolic pathway for PCL degradation begins with the hydrolysis of ester bonds outside the cell, resulting in the formation of 6-hydroxyhexanoic acid and related short-chain hydroxy acids. These intermediates are then oxidized to dicarboxylic acid derivatives and directed to the β-oxidation pathway, producing acetyl-CoA, which enters the tricarboxylic acid (TCA) cycle. Short-chain hydroxy acids and carboxylic acids were also detected during PCL biodegradation. The stoichiometric end products of complete mineralization are CO_2_, H_2_O, and microbial biomass [73].

## 4. Origins of Microbial Plastic Degradation Potential

### 4.1. Microbes from Contaminated Ecosystems

Ecosystems that are exposed to plastics over long periods of time provide a natural selective habitat (an environment conducive to the survival of organisms with specific characteristics) for microorganisms capable of using polymers as a source of carbon and energy. This phenomenon is referred to as the “plastisphere”—a unique ecological niche comprising communities of microorganisms colonizing plastic surfaces, clearly differing in composition from the microbiota of the surrounding environment [74,75]. Long-term exposure to synthetic polymers exerts selective pressure that promotes the development of strains producing enzymes capable of attacking chemical bonds in polymer chains [53,76,77].

Studies of landfills, industrial sites, and sewage sludge have revealed the presence of numerous bacterial and fungal strains exhibiting depolymerization activity. In a report by Hussein et al. (2015), 169 bacterial isolates were isolated from municipal waste landfills in Baghdad, of which 42 showed a high ability to degrade low-density polyethylene (LDPE), with particular emphasis on the strains *Pseudomonas fluorescens*, *Pseudomonas aeruginosa* and *Acinetobacter ursingii* [78]. Similar observations are reported by Grijalva et al. (2025), who isolated strains of *Bacillus cereus*, *Acinetobacter baumannii*, and *Pseudomonas otitidis* from landfills capable of degrading polybutyrate (PBS—biodegradable polymer), causing visible damage to the surface of polymer films and attacking the ester bonds in the polymer structure [79]. Particularly significant discovery was the isolation by Yoshida et al. (2016) from a PET bottle recycling plant in Sakai (Japan) of the bacterium *Ideonella sakaiensis* 201-F6, which is capable of utilizing polyethylene terephthalate (PET) as its sole source of carbon and energy [53]. This strain produces two enzymes: PETase (PET hydrolase, an enzyme that breaks the ester bonds in the PET chain) and MHETase (MHET hydrolase, an enzyme that converts the intermediate degradation product into monomers: terephthalic acid and ethylene glycol) [53,80,81]. This discovery led to intensive research into the enzymatic degradation of polyesters, studies showed that *I. sakaiensis* was capable of degrading up to 96% of commercial transparent PET materials from food packaging within seven weeks of incubation [82].

Municipal wastewater and sewage sludge environments are equally promising sources of plastic-degrading microorganisms. Study by Ali et al. (2023) showed that the strains *Pseudomonas* sp. SH5B and *Pseudomonas aeruginosa* SH6B isolated from municipal wastewater achieved a 25% weight loss of plastic balloons after 120 days of incubation, with FTIR spectroscopic analysis (Fourier transform infrared spectroscopy) confirmed the formation of new chemical bonds and a change in the polymer structure [83]. Mohanan et al. (2020) documented the isolation of strains capable of degrading unmodified polyethylene from numerous contaminated environments, including oil-contaminated soils, sewage sludge, landfills, and agricultural film waste [84]. Latest studies confirm that regardless of the type of polymer substrate used in enriching cultures, similar bacterial types are isolated from landfill soils, sewage sludge, and river waters, with a predominance of *Pseudomonadota* (64.86%), *Bacteroidota* (16.22%), and *Actinomycetota* (13.51%), suggesting the existence of universal taxa with broad degradation potential [20].

Mechanism of selection pressure in ecosystems polluted with plastics involves several stages: initial colonization of the polymer surface by pioneer microorganisms, followed by a selection phase, during which organisms capable of degrading the polymer become enriched, and finally a succession phase, in which degraders are gradually replaced by organisms that utilize the products of decomposition, known as cross-feeders [18,85]. Higher selection pressure observed in the plastisphere of biodegradable polymers (e.g., PLA) compared to polyethylene may result from the greater availability of degradation products as carbon sources for microorganisms [85].

### 4.2. Soil Microbiota

Soil, as a heterogeneous ecosystem, is characterized by exceptional microbiological biodiversity. Soil microorganisms, due to their evolutionary adaptation to the breakdown of complex biopolymers (natural polymers of biological origin, e.g., lignin or chitin), exhibit significant potential for the degradation of synthetic polyesters. Of particular importance is the fact that ligninolytic enzymes, such as laccases, lignin peroxidases (LiP), and manganese-dependent peroxidases (MnP), have the ability to attack chemical bonds in synthetic polymers with a structure similar to lignin, including polyethylene (PE) and polystyrene (PS) [84,86]. Strains of the genera *Bacillus*, *Pseudomonas*, *Streptomyces*, and *Rhodococcus* have been repeatedly identified as organisms capable of initiating the biodegradation of various polymers [84,87]. Research by Li et al. (2022) showed that *Bacillus subtilis* ATCC6051 and *Bacillus licheniformis* ATCC14580, strains widely distributed in natural soils, cause a loss of LDPE (low-density polyethylene) by 3.49% and 2.83%, respectively, over 30 days of incubation, with visible structural changes on the surface (cracks, depressions, roughness) observed under an optical microscope [88]. The depolymerization activity of these strains was confirmed by FTIR spectroscopy, which showed the formation of unsaturated bonds and carbonyl and hydroxyl groups. In recent studies, Khampratueng et al. (2024) isolated the strains *Bacillus* sp. AS3 and *Sphingobacterium* sp. AS8 from landfills, which achieved a weight loss of LDPE film of 3.06% and 2.01%, respectively, within four weeks, while producing esterase with an activity of 0.608 U/mL [48].

The genus *Pseudomonas* is one of the most intensively studied taxa (systematic units) in the context of plastic biodegradation. The strains *Pseudomonas aeruginosa*, *P. fluorescens*, and *P. putida* show the ability to degrade both polyolefins (PE, PP) and polyesters (PET, PUR) [84,89]. Of particular importance is the presence in these bacteria of alkB genes encoding alkane monooxygenases, integral membrane non-heme diiron metalloenzymes whose primary catalytic function is the terminal hydroxylation of saturated straight-chain alkanes [90,91]. The prototypical AlkB from *Pseudomonas oleovorans* (GPo1) and its close homologs are active on n-alkanes containing 5–12 carbon atoms (with optimal activity on C7-C8), whereas AlkB variants carrying a smaller hydrophobic residue in the substrate-channel restriction site can metabolize longer alkanes, typically C12 and above [90,92]. Although AlkB shows exclusive terminal C-H bond selectivity on saturated chains, it is not strictly limited to saturated substrates: AlkB enzymes also catalyze the epoxidation of terminal alkenes (compounds containing a C=C double bond), and engineered variants have been used to derivatize alkenes to the corresponding epoxides, in addition to producing alcohols, aldehydes and carboxylic acids [91,92]. Bacterial consortia containing *Pseudomonas* and *Bacillus* species exhibit synergistic activity in PET degradation, with complete conversion of BHET (bis(2-hydroxyethyl) terephthalate, an intermediate product of PET degradation) to metabolically useful monomers: terephthalic acid (TPA) and ethylene glycol [89].

Actinomycetes, in particular the genera *Streptomyces* and *Rhodococcus*, are another group with documented degradation potential. *Streptomyces* sp. produces proteins from the LCP family (latex clearing protein), which are active against polyethylene [93]. *Rhodococcus ruber* and *R. jostii* RHA1, strains originally identified as biodegrading polychlorinated biphenyls (PCBs, toxic organic compounds), exhibit the ability to colonize PE films and induce changes in the molecular weight of the polymer, with the participation of multi-copper laccases [94]. Metagenomic studies of LDPE-enriched consortia identified *Actinobacteria* and *Proteobacteria* as the dominant bacterial types, with genera including *Mycobacterium*, *Gordonia*, *Nocardia*, and *Amycolatopsis* [20,95].

Fungi are an especially effective group of organisms capable of degrading plastics due to their ability to secrete extracellular enzymes with a broad substrate spectrum and tolerance to stressful conditions, as well as limited availability of nutrients [96,97,98,99,100]. *Aspergillus* and *Penicillium* species dominate the soil microbiome and have been identified as the most frequently isolated taxa capable of degrading both biodegradable and conventional polymers [51,52,53]. More than 15 species of the genus *Aspergillus* have been documented to degrade LDPE, including *A. niger*, *A. flavus*, *A. fumigatus*, *A. terreus*, *A. oryzae*, and *A. nomius* [99,101,102]. *Aspergillus tubingensis* also has proven the ability to colonize plastic surfaces and produce enzymes that break the chemical bonds between polymer molecules [103].

The mechanism of polymer degradation by fungi involves several stages: initial attachment of fungal hyphae to the hydrophobic surface of the polymer via hydrophobin, followed by biofilm formation and secretion of specific polymer-degrading enzymes [103,104]. The most important fungal enzymes involved in the biodegradation of plastics include: cutinases (EC 3.1.1.74, enzymes that hydrolyze cutin, a natural plant polyester, exhibiting activity against PET and other synthetic polyesters), lipases (EC 3.1.1.3, triacylglycerol hydrolases capable of hydrolyzing ester bonds), laccases (EC 1.10.3.2, polycopper oxidases catalyzing single-electron oxidation of phenolic substrates), and peroxidases (enzymes using hydrogen peroxide to oxidize substrates) [10,105].

Research by Ibrahim et al. (2024) showed that strains of *Fusarium*, *Penicillium*, *Botryotinia cinerea*, and *Trichoderma* have high potential for degrading polyurethane (PUR), rubber, and polyethylene (PE), consuming over 90% of oxygen within 14 days while producing 300–500 ppm of CO_2_, without the need for preliminary treatment of plastics and without the addition of sugars [106]. White rot fungi (white-rot fungi of the genera *Phanerochaete* and *Pleurotus*, producing a full set of ligninolytic enzymes (lignin peroxidase, manganese-dependent peroxidase, and laccase), show the ability to degrade polyethylene through mechanisms analogous to lignin decomposition. The degradation process leads to changes in the chemical structure of polyethylene, including the formation of polar functional groups such as carbonyl (C=O), carboxyl (O=C-OH), and hydroxyl (-OH) [96,107]. The evolutionary relationship between degradation mechanisms is confirmed by the observation that polystyrene (PS), due to its aromatic structure partially similar to lignin, is effectively degraded by the intestinal microorganisms of *Tenebrio molitor* (mealworm beetle) larvae, whose microbiome is adapted to the decomposition of lignin-rich plant materials [44]. Other study showed that *Aspergillus niger* and *Phanerochaete chrysosporium* isolated from garbage soil are capable of biodegrading LDPE, with the highest efficiency (approximately 40% after 20 days) achieved in Czapek-Dox medium, and *A. niger* exhibiting greater degradative activity. SEM, FTIR, XRD, and DSC analyses confirmed degradation through morphological, chemical, and structural changes in the polymer, indicating chain breakdown and modification of LDPE crystallinity [108].

Contemporary research emphasizes the importance of microbial consortia in the biodegradation of plastics. The chemical and structural complexity of synthetic polymers means that a single microorganism is rarely capable of complete mineralization of a polymer [109]. Similarly, co-cultures of *Bacillus* sp. and *Priestia* sp. achieved higher degradation of LDPE film (0.667% weight loss after 48 days) compared to single isolates, with higher biofilm and esterase production [110]. Bacterial-fungal consortia exhibit complementary enzymatic capabilities: bacteria often initiate the oxidation of polymer chains, while fungi ensure deeper penetration of the substrate through hyphal growth and the production of thermostable hydrolases. For example, Salinas et al. 2024 demonstrated that a bacterial-fungal consortium composed of *Bacillus subtilis* RBM2, *Fusarium oxysporum* RHM1, and *Alternaria alternata* RHM4 achieved approximately 18% weight loss of recycled LDPE within only 30 days, substantially outperforming monocultures and confirming that strategic consortia design based on complementary esterase and ligninase profiles significantly enhances polyolefin biodegradation [111]. In parallel, enriched microbial consortia derived from plastic-contaminated waste environments have demonstrated superior polymer-degrading activity compared to individual isolates, as members exchange metabolic intermediates and share the removal of toxic by-products, thereby sustaining prolonged synergistic degradation activity [112].

### 4.3. Compost Microbiota

The composting environment is a very promising reservoir of microorganisms with biodegradation potential. Composting is a dynamic thermal process that includes a mesophilic phase (25–40 °C), a thermophilic phase (55–65 °C, and in some cases up to 70 °C), and a maturation phase with a gradual decrease in temperature [55]. The elevated temperature in the thermophilic phase, the diverse substrate composition, and the intense microbial activity create unique selection conditions that favor the growth of thermophilic microorganisms producing thermostable hydrolytic enzymes. This is because temperature is a very important parameter determining the efficiency of enzymatic degradation of plastics, especially polyesters. The glass transition temperature (Tg, the temperature above which the polymer becomes elastic and more susceptible to enzymatic attack) for semi-crystalline PET is around 70 °C, while for amorphous PLA it ranges from 50–70 °C [16,110,111,112]. Research also shows that thermophilic conditions above 50 °C significantly improve PLA biodegradation compared to lower temperatures [113,114,115,116,117], and preliminary thermal treatment of PLA can significantly enhance subsequent biodegradation [118]. Therefore, thermophilic compost microbiomes are a promising source of enzymes with high activity and stability. The most effective PET-degrading enzyme described to date is leaf-branch compost cutinase (LCC), isolated by metagenomic methods from bacteria colonizing compost [52]. Enzyme exhibits optimal hydrolytic activity towards PET at 50 °C in addition to the ability to decompose PET completely into terephthalic acid (TPA) and ethylene glycol [52].

Thermophilic actinomycetes isolated from compost constitute the dominant group of microorganisms initiating polyester degradation [119]. The genera *Thermobifida*, *Thermomonospora*, and *Thermobispora* have repeatedly been identified as highly effective degraders of aliphatic-aromatic polymers. *Thermomonospora fusca* (now reclassified as *Thermobifida fusca*), the first thermophilic actinomycete to be described, has been shown to produce polyesterase active against aromatic polyesters, including PET [120]. The strains *Thermobifida fusca*, *T. alba*, and *T. cellulosilytica* produce cutinases with a broad substrate spectrum, capable of hydrolyzing both aliphatic polyesters (PCL—polycaprolactone) and aromatic copolyesters (PET, PTT—poly(trimethylene terephthalate)) [84,121]. Recent studies have shown that *Thermobifida fusca* cutinase (TfCut2) is a carboxylesterase that degrades both PET and its hydrolysis intermediates (OET, BHET, MHET) into terephthalic acid (TPA) [122]. Recently, two strains: *Pseudomonas* G1 and *Kocuria* G2 were isolated from the thermophilic phase of compost; together, they form a co-culture system that has been described for the first time as effectively degrading PLA, PBAT, and PLA/PBAT-ST20 films. After 15 days of compost bioaugmentation, the co-culture system achieved a degradation rate of PLA/PBAT-ST20 films of 72.14 ± 2.1% by weight, and the synergy between the two strains significantly increased the secretion and activity of proteases and lipases [116]. On the other hand, complete degradation of PLA beverage cups was observed after 15 days in soil with sewage sludge from a dairy under thermophilic conditions when PLA was pretreated with UV radiation [123]. Under thermophilic anaerobic fermentation conditions (55–58 °C), a significantly higher conversion of PLA to methane was observed than under mesophilic conditions; for untreated and pretreated PLA materials, a maximum methane yield of 453 L/kg of volatile solids (VS) was achieved, which corresponds to nearly 100% of the theoretical methane yield from PLA, with hydrothermal treatment reducing the time to reach maximum methane production by more than two weeks compared to untreated PLA, and methane being produced at a rate nearly 10% higher (8.35 vs. 7.79 L/(kg VS·d) [124]. It was also demonstrated that under thermophilic conditions (58 °C), thermoplastic starch, PHB, and PLA achieve a high level of biodegradation in a relatively short time (<100 days), whereas PBS, PBAT, and PCL are not converted to methane under these conditions, and a key role in PLA degradation is played by lactate-utilizing syntrophic bacteria of the genera *Moorella* and *Tepidimicrobium* [125]. Furthermore, two highly effective strains capable of degrading post-consumer PLA packaging waste were isolated from compost samples: *Bacillus* sp. SNRUSAC1 and *Priestia aryabhattai* SNRUSAC3, with this being the first report of PLA degradation by the species *P. aryabhattai*; after optimization of culture conditions, these strains achieved 62.06% and 57.61% dry weight loss, respectively, in just 30 days [126]. Recently, using functional enrichment cultures, 14 unique microorganisms capable of degrading PLA and PBAT were isolated, and using a computational enzyme discovery pipeline, a set of 97 enzymes was functionally characterized, identifying three active PLA-degrading enzymes [127]; complementarily, it was confirmed that wastewater from municipal treatment plants constitutes a reservoir of PLA-degrading enzymes, with the order Bacillales dominating under moderately thermophilic conditions (50 °C) alongside an increase in the abundance of genes encoding potential PLA-degrading enzymes, whereas under mesophilic conditions (37 °C), despite ongoing PLA degradation, no enrichment of known depolymerases was observed, suggesting the presence of new enzymes and/or degradation pathways [128].

Latest research by Salinas et al. (2025) has shown that ligninolytic microorganisms isolated from the composting process of agricultural waste are a promising source of plastic-degrading consortia [55]. The genera *Bacillus*, *Pseudomonas*, *Fusarium*, *Aspergillus*, *Scedosporium*, and *Pseudallescheria* exhibited a wide range of enzymatic activities related to plastic biodegradation. Consortia combining *Bacillus subtilis* RBM2 with *Fusarium oxysporum* RHM1 increased the efficiency of polyethylene degradation, while the bacterial-bacterial combinations of *Pseudomonas aeruginosa* RBM21 and *Bacillus subtilis* RBM2 showed a broad spectrum of degradation including linear low-density polyethylene (LLDPE), PET, and polystyrene (PS) [55].

### 4.4. Uncultured Microbiota

A substantial fraction of environmental microorganisms cannot be recovered under standard laboratory conditions, which means that culture-dependent screening systematically overlooks the majority of potentially valuable plastic-degrading taxa and their enzymes. Metagenomic approaches, both functional screening of environmental DNA libraries and sequence-based in silico mining of assembled metagenomes, have emerged as the principal strategy for accessing this hidden enzymatic reservoir, and recent large-scale studies have revealed just how vast it is. Using hidden Markov models built from experimentally verified plastic-degrading enzymes, Zrimec et al. mined ocean and soil metagenomes across 236 sampling locations worldwide and compiled a catalogue of more than 30,000 non-redundant enzyme homologues with the potential to degrade 10 different plastic types, with nearly 60% of the identified sequences not mapping to any previously known enzyme class [129]. The study thus revealed a significant correlation between the abundance of these newly identified enzymes in both marine and soil environments and trends in plastic pollution in the ocean and on land. Synergistic biodegradation of the aromatic-aliphatic copolyester PBAT has been dissected by combined metagenomic and metaproteomic profiling of a marine microbial consortium, in which six PETase-type and four MHETase-type enzymes were jointly identified as drivers of depolymerization. A consortium of marine microorganisms, mainly from *Marinobacter* and *Alphaproteobacteria*, exhibits synergistic biodegradation of an aromatic-aliphatic copolyester (PBAT), using it as the sole carbon source and achieving significant conversion to CO_2_ and biomass within approximately 15 days [130]. Shotgun metagenomics of alpine plastisphere soils has further enabled the discovery of a novel hydrolase-type esterase from an uncultured *Caballeronia* sp., illustrating that metagenomic mining can recover active plastic-degrading enzymes even from cold, oligotrophic environments. In this study, the metagenomes of the plastosphere and bulk soil were screened using differential abundance analysis and similarity-based screening against databases of known plastic-degrading genes. Candidate genes were selected based on a high probability of signal peptides for extracellular export and strong confidence in their functional domains. This rigorous screening process in silico resulted in a final list of nine candidate genes [131]. In mangrove soils subjected to enrichment selection, metagenome-assembled genomes revealed *Mangrovimarina plasticivorans* gen. nov., sp. nov., carrying putative novel monohydroxyethyl terephthalate hydrolases and expanding the phylogenetic diversity of known PET-active microorganisms. Phylogenomic analysis, including a Genome BLAST Distance Phylogeny (GBDP) tree and associated single-copy protein genes, confirmed that MAG9_P4 and MAG11A_P8 belong to a new genus closely related to *Nitratireductor mangrovi* SY7 [2]. Most recently, metagenomic screening of hydrothermally impacted deep-sea sediments from the Guaymas Basin led to the discovery of GuaPA, which is recognized as the first archaeal PETase, from an uncultured Bathyarchaeia, and exhibits optimal activity at 60 °C, a melting temperature of 84 °C, and releases 3–5 mM of terephthalic acid and MHET from low-crystallinity PET within 48 h. This study identified a total of 300 unique candidate enzymes, 22 of which were selected based on their thermophilic potential, phylogenetic novelty, and structural similarity to known PETases [23]. All in all, these advances make metagenomics an indispensable fourth source of origins, capable of complementing studies of the microbiota in contaminated sites, soil, and compost. Furthermore, as demonstrated above, it can be effective in the search for enzymes that degrade plastics, which is particularly important in the case of biocatalysts derived from extremophilic, slow-growing, or otherwise uncultivable taxa.

A consolidated overview of the representative isolates and enzymes discussed across Section 3.1, Section 3.2, Section 3.3 and Section 3.4, together with their environmental origins, reported degradation parameters and experimental conditions, is provided in Supplementary Table S1.

### 4.5. Mechanisms of Consortium Synergy

Consistent observations emerge for all four sources discussed above: isolates from the plastisphere, soil microbiota, compost communities, and uncultivable taxa identified via metagenomics: microbial consortia outperform single-strain cultures in polymer transformation, often by a significant margin. The mechanistic basis for this advantage lies in three interrelated processes that occur at different organizational levels: metabolic cross-feeding, cooperation via biofilm, and population-level coordination through quorum sensing.

Metabolic cross-feeding refers to the exchange of partially oxidized intermediates and growth factors among consortium members, whereby the by-products of one species serve as substrates for another. This mechanism is particularly relevant to plastic biodegradation because the initial oxidation of polyolefins, polystyrene, and polyesters generates intermediates such as alcohols, aldehydes, ketones, mono- and dicarboxylic acids, styrene oxide, phenylacetic acid, and short-chain hydroxy acids that frequently accumulate to growth-inhibitory concentrations in monocultures. Co-occurring scavenger species capable of channeling these intermediates into central metabolic routes such as β-oxidation, the β-ketoadipate pathway, and the TCA cycle, relieve end-product inhibition and sustain the activity of primary depolymerizers. A recent experimental dissection of this principle was provided by Tarara et al. (2025), who showed that a soil-derived consortium combining *Pseudomonas* and *Bacillus* species degraded UV-pretreated PE film and powder by 7% and 13% of mass, respectively, within 30 days as the sole carbon source, with GC/MS detecting the alkane- and carboxylic acid-derived intermediates expected from cooperative oxidative metabolism, and with the cytochrome P450 reductase CYP102 A5 from *Bacillus thuringiensis* identified as one of the molecular contributors to PE oxidation in the consortium [132]. Cross-feeding additionally encompasses the exchange of vitamins, amino acids, and electron acceptors that compensates for biosynthetic auxotrophies frequently observed among efficient plastic-colonizing specialists, and it is increasingly viewed as a rational design principle for engineered synthetic microbial consortia targeting recalcitrant polymers such as PET. This phenomenon helps, among others, prevent the accumulation of intermediate products, such as mono(2-hydroxyethyl) terephthalate (MHET), which can inhibit the initial hydrolysis of PET [133].

Biofilm formation provides a second layer of synergistic enhancement. Hydrophobic polymer surfaces such as PE, PP, and PS are colonized through the secretion of biosurfactants, hydrophobins (in fungi), and an extracellular polymeric substance (EPS) matrix composed of exopolysaccharides, structural proteins, and extracellular DNA, which together reduce interfacial hydrophobicity, increase the effective contact area between cells and substrate. Furthermore, activity of oxidative enzymes may promote bacterial adhesion by increasing surface roughness (e.g., by creating pits, cracks, and other irregularities) or by altering the surface properties of petropolymers from hydrophobic to hydrophilic, thereby further enhancing microbial colonization [134]. The matrix additionally retains reactive oxygen species generated by laccases and peroxidases in close proximity to the polymer, protects cells from desiccation and from accumulating toxic intermediates, and creates spatial gradients of oxygen and pH that favour the parallel coexistence of aerobic primary degraders at the biofilm surface and microaerophilic or anaerobic scavengers in deeper layers. The functional consequences of this stratification have been demonstrated by Li et al. (2025) using experimentally evolved PE-degrading bacterial biofilms, in which *Stutzerimonas stutzeri* emerged as the dominant degrader expressing the enzymes responsible for PE oxidation, while two co-evolved community members specialized in the secretion of extracellular polysaccharides supporting biofilm architecture; (meta)transcriptomic profiling confirmed asymmetric cross-feeding and spatial partitioning of biofilm space as the basis of synergistic activity [135].

In bacterial-fungal consortia, an analogous division of labor operates at a larger spatial scale, with fungal hyphae mechanically penetrating the polymer and creating fissures that bacteria subsequently colonize, generating a positive feedback between physical disruption and enzymatic attack. Direct experimental support for this mechanism comes from several recent studies. Jia et al. (2021) showed that a co-culture of *Pseudomonas mendocina* and the filamentous fungus *Actinomucor elegans* degraded PLA/PBAT films by 18.95 wt% within five days [136]. This rate substantially exceeded those of the bacterial (12.94 wt%) and fungal (9.27 wt%) monocultures [136]. A complementary demonstration on polyolefins was provided by Alidoosti et al. (2025), who studied a fungal-bacterial co-culture of *Bacillus velezensis* EBL50 and *Sarocladium strictum* EBL60 acting on LDPE microplastics [137]. The co-culture achieved 26.3% mass loss after 60 days which is approximately twice the rate of the fungal monoculture (13.2%) and four times that of the bacterial monoculture (6.8%). Crucially, the half-life of LDPE was reduced from 602 to 134 days. SEM, FTIR, and GC-MS analyses confirmed surface erosion, oxidative chemical modification, and the production of C14-C24 alkanes, consistent with combined oxidoreductase activity (laccases, peroxidases, cytochrome P450) contributed by both partners [137]. The role of fungal hyphae in providing a physical scaffold for bacterial colonization has been further confirmed by Cao et al. (2022) for microbial consortia degrading complex pollutants, supporting the view that hyphae serve as both a mechanical disruptor of polymer surfaces and a substratum that increases bacterial access to otherwise inaccessible regions of the substrate [138].

Quorum sensing, cell-density-dependent gene regulation mediated principally by *N*-acyl homoserine lactones (AHLs) in Gram-negative bacteria and by autoinducing peptides in Gram-positive bacteria, has long been recognized as a regulator of biofilm maturation, EPS production, biosurfactant secretion, and several extracellular hydrolases at the population level [139]. Direct evidence that this regulatory layer is operative in the plastisphere has only recently emerged. Wang et al. (2026) analyzed global plastisphere metagenomes and reported a significant enrichment of QS- and biofilm-formation genes, with a particularly strong signal for AHL-mediated QS; complementary microfluidic and tubular-column experiments demonstrated that exogenous AHL actively promoted plastisphere formation, biomass accumulation, and EPS production on microplastics, whereas the application of a quorum-quenching enzyme (AHL acylase) suppressed all three processes [140]. At the level of single-strain physiology, primary PET degradation products themselves were shown to feed back into intracellular signaling: in *Vibrio gazogenes* DSM 21264, BHET and terephthalic acid modulated c-di-GMP, cAMP, and QS-dependent gene expression, linking polymer-derived metabolites to the regulation of biofilm formation and PET hydrolase secretion [141]. Despite these advances, the role of the quorum sensing (QS) system in regulating the activity of specific plastic-degrading enzymes, as well as the extent to which interspecies QS communication coordinates the division of labor within mixed consortia, remains poorly understood. Furthermore, rational manipulation of signaling via AHL and peptides represents a new avenue for designing more efficient, natural, and synthetic plastic-degrading communities.

## 5. Current Challenges

The degradation of plastics using native microorganisms presents many challenges. Above all, these are low efficiency and long degradation times. Most of the environmental isolates described achieve only a few percent loss of polymer mass over weeks or months of incubation. For example, the strains *Bacillus subtilis* and *B. licheniformis* cause a loss of LDPE mass of 2–3% within 30 days. Such low values are far from the requirements of industrial biological recycling, which would require complete mineralization within hours or days. Most of the environmental isolates described achieve only a few percent loss of polymer mass over weeks or months of incubation (representative values are compiled in Supplementary Table S1.

On the other hand, polymers with high mechanical strength are consistently structurally resistant to enzyme action. Polyolefins (PE, PP) do not have ester bonds susceptible to hydrolysis, which requires the use of slower oxidative enzymes.

One of the strategies for increasing the susceptibility of plastics to biological systems is pre-treatment and the use of thermophilic conditions. The effective biodegradation of many polymers requires preliminary abiotic weathering (UV, thermal oxidation), which leads to changes in the arrangement of functional groups, e.g., by bringing carbonyl and hydroxyl groups to the surface. In addition, the glass transition temperature of PET (~70 °C) and PLA (50–70 °C) means that effective enzymatic degradation occurs mainly under thermophilic conditions (>50 °C), which limits their use in natural environments with lower temperatures.

Another problem is the chemical complexity of synthetic polymers, which means that a single microorganism is rarely capable of complete mineralization. Bacterial-fungal consortia with complementary enzymatic capabilities are necessary, but this complicates the optimization and control of biotechnological processes. At the same time, mixed plastic waste requires the simultaneous action of many different degradation mechanisms.

There are high hopes for genetically engineered strains, but despite significant advances in enzyme modification (PETase, MHETase, cutinase), including increased thermostability and catalytic activity, the practical application of GMO strains faces significant regulatory, biosafety, and scalability obstacles. Therefore, the search for effective environmental isolates remains a more viable path to industrial applications.

Another significant problem directly affecting the applicability of enzyme-based biological methods is the scalability of laboratory results to an industrial scale. There is also a lack of standardized degradation activity tests, which makes it difficult to compare strains and enzymes between different studies. There is also a lack of standardized degradation activity tests, which makes it difficult to compare strains and enzymes between different studies. In particular, the limited adoption of respirometric protocols (Sturm test, ISO 14855, ASTM D5338) and isotope-tracing approaches using ^13^C- or ^14^C-labeled substrates, which remain the only definitive methods to distinguish complete mineralization from polymer fragmentation, surface erosion, or partial assimilation, represents a major methodological gap that hampers reliable cross-study comparison and impedes regulatory acceptance of microbial bioremediation technologies. Another key challenge is the integration of depolymerization with monomer valorization pathways (bio-upcycling), which is necessary to ensure the economic viability of processes within a circular economy.

In summary, the current challenges are that the main barriers to the biodegradation of plastics are both biological (low enzymatic activity, lack of universal degraders) and technological (temperature requirements, need for pre-treatment, scalability). Further progress requires the simultaneous development of screening methods for environmental isolates, the optimization of microbial consortia, and the construction of integrated pathways combining depolymerization with product valorization.

## 6. Conclusions

This review provides a comprehensive overview of the current state of knowledge on plastic-degrading microorganisms, enzymatic mechanisms, and the environments from which the most promising isolates originate. Biodegradation pathways differ significantly depending on the polymer class: polyesters, such as PET and PLA, undergo hydrolytic cleavage of ester bonds by PETases, cutinases, and related hydrolases, while polyolefins (PE, PP) and polystyrene (PS) require oxidative attack by laccases, peroxidases, and alkane monooxygenases, followed by β-oxidation of the resulting fragments. This diversity of mechanisms highlights the need to tailor enzymatic strategies to each type of polymer.

Three main environmental reservoirs of plastic-degrading microorganisms have been identified. Contaminated sites and the plastisphere are home to diverse bacterial communities, mainly species of *Pseudomonas*, *Bacillus*, and *Ideonella*, selected by long-term exposure to synthetic polymers. Soil microbiota provides ligninolytic fungi (*Aspergillus*, *Penicillium*, white rot species) and actinomycetes with a broad spectrum of hydrolytic and oxidizing enzymes. Composting environments, especially in the thermophilic phase, produce thermostable enzymes such as cutinase from leaf and branch compost (LCC), currently the most effective PET hydrolase described, and thermophilic actinomycetes of the genera *Thermobifida* and *Thermomonospora* with activity profiles of industrial relevance.

Despite these advances, significant challenges remain. The degradation rate of most environmental isolates is still far below industrial requirements, complete mineralization by single organisms is rarely achieved, and highly crystalline or non-hydrolyzable polymers remain difficult to degrade. Furthermore, the transfer of laboratory results to scalable bioprocesses is hampered by the lack of standard activity tests and the difficulty of maintaining complex microbial consortia under controlled conditions.

Future efforts should therefore focus on three converging priorities: (i) systematic environmental research programs combined with standardized degradation benchmarks, (ii) the development of robust microbial consortia and thermostable enzyme cocktails optimized for closed bioreactor systems, and (iii) the integration of depolymerization with monomer valorization pathways to enable economically viable bio-upcycling within a circular economy. The remarkable metabolic diversity already documented in contaminated sites, soils, and composting environments provides a solid foundation for achieving these goals.

## Data Availability

No new data were created or analyzed in this study.

## Supplementary Materials

The following supporting information can be downloaded at: https://www.mdpi.com/article/10.3390/molecules31101638/s1, Table S1: Representative environmental isolates and enzymes for plastic biodegradation: microbial source, polymer substrate, degradation efficiency and experimental conditions [32,41,43,46,48,52,53,62,78,82,83,89,93,94,101,106,109,111,120,123,142,143,144,145,146].
