# Supplementary material for: Plastic-Degrading Microorganisms: Biodegradation Pathways and Habitat Origins
Source: Molecules. 2026 May 13;31(10):1638. doi: 10.3390/molecules31101638 (PMC13209321; doi:10.3390/molecules31101638)
Supplement: Supplementary file 1 [file molecules-31-01638-s001.zip › molecules-4267453-supplementary.pdf]

Supplementary Table 1. Representative environmental isolates and enzymes for plastic biodegradation: microbial source, polymer substrate, degradation efficiency and experimental conditions.

| Polymer | Microorganism / Enzyme | Origin / Habitat | Degradation result | Experimental conditions | Ref. |
|---|---|---|---|---|---|
| LDPE | *Bacillus subtilis* ATCC6051 and *Bacillus licheniformis* ATCC14580 | Soil microbiota (cosmopolitan natural soils) | 3.49 % and 2.83 % mass loss; FTIR: formation of unsaturated bonds, carbonyl and hydroxyl groups; surface cracks, depressions and roughness (optical microscopy) | 30 days incubation; no abiotic pretreatment reported | [1] |
| | *Bacillus* sp. AS3 and *Sphingobacterium* sp. AS8 | Municipal waste landfill (dumping site community) | 3.06 % and 2.01 % LDPE film mass loss; extracellular esterase activity 0.608 U/mL | 4 weeks incubation; no abiotic pretreatment reported | [1] |
| | *Pseudomonas fluorescens*, *Pseudomonas aeruginosa* and *Acinetobacter ursingii* | Municipal waste landfill, Baghdad | 42 of 169 isolates showed high LDPE-degrading capability (qualitative screening) | Standard mineral-medium screening with LDPE as sole carbon source | [2] |
| | *Pseudomonas* sp. SH5B and *Pseudomonas aeruginosa* SH6B | Municipal wastewater | 25 % mass loss of plastic balloons; FTIR confirmed new chemical bonds and altered polymer structure | 120 days incubation | [3] |
| | *Lysinibacillus xylanilyticus* + *Aspergillus niger* (bacterial–fungal consortium) | Soil | Higher LDPE degradation efficiency than corresponding single-species cultures | Soil-microcosm assay; mesophilic conditions | [4] |
| | *Bacillus* sp. + *Priestia* sp. (co-culture) | Landfill | 0.667 % LDPE film mass loss; enhanced biofilm formation and esterase production vs. single isolates | 48 days incubation | [5] |

| Polymer | Microorganism / Enzyme | Origin / Habitat | Degradation result | Experimental conditions | Ref. |
|---|---|---|---|---|---|
| PE (LDPE / general PE) | *Rhodococcus* sp. (naturally weathered plastic enrichment) | Naturally weathered plastic waste enrichment | PE chain oxidation; intermediates incl. alcohols, aldehydes, ketones, mono- and dicarboxylic acids, palmitic acid (medium-chain fatty acid) | Aerobic enrichment culture | [6] |
| PE | *Streptomyces* sp. K30 (latex clearing protein, LCP family) | Soil actinomycete | PE-degrading activity demonstrated; activity further enhanced as anchor-peptide–LCP fusion protein | *In vitro* and whole-cell assays | [7] |
| | *Rhodococcus ruber* and *R. jostii* RHA1 (multi-copper laccases) | Soil; originally identified as PCB-degraders | PE film colonization; reduction of PE molecular weight | Mesophilic, aerobic incubation | [8] |
| PE, PUR, rubber | *Fusarium*, *Penicillium*, *Botryotinia cinerea*, *Trichoderma* | Soil-derived fungal isolates | > 90 % $O_2$ consumption; 300–500 ppm $CO_2$ produced | 14 days incubation; no plastic pretreatment, no added sugars | [9] |
| PS | *Stenotrophomonas maltophilia* sp. ZSL2493 (Kmo, Hpd) | Gut of *Tenebrio molitor* larvae | 14.7 % PS biodegradation by parent strain; recombinant Kmo: 1.37 µg/mL styrene; recombinant Hpd: 0.95 µg/mL styrene | 30 days incubation; mesophilic; *in vitro* for recombinant enzymes | [10] |
| | *Exiguobacterium* sp. RIT594 | Environmental isolate | Biofilm formation, surface indentations, reduced hydrophobicity, appearance of carboxyl/hydroxyl groups; FTIR-confirmed dearomatization (ring-cleaving dioxygenase pathway) | Aerobic; molecular oxygen required | [11] |
| | *Microbacterium esteraromaticum* SW3 | Soil | PS used as sole carbon source; manganese peroxidase and lipase activity influencing polymer conversion | Aerobic, mesophilic incubation | [12] |

| Polymer | Microorganism / Enzyme | Origin / Habitat | Degradation result | Experimental conditions | Ref. |
|---|---|---|---|---|---|
| | *Tenebrio molitor*, *Zophobas atratus*, *Galleria mellonella* (gut microbiota) | Insect gut consortia | Rapid *in vivo* PS depolymerization and mineralization; $^{13}C$-labelling confirmed C incorporation into biomass and $CO_2$ release; antibiotic-mediated gut bacteria inhibition abolished both depolymerization and mineralization | *In vivo* larval feeding; ambient temperature | [13] |
| PET | *Ideonella sakaiensis* 201-F6 (PETase + MHETase) | PET-bottle recycling plant, Sakai (Japan) | Up to 96 % degradation of commercial transparent PET food-packaging material | 7 weeks incubation; mesophilic (~30 °C); low-crystallinity PET | [14,15] |
| | Leaf-branch compost cutinase (LCC); uncultured thermophilic actinobacterium (~57.4 % identity to T. fusca cutinase, ~59.7 % to T. curvata lipase) | Functional metagenomic screening of fosmid library from leaf-branch compost (~67 °C) | Optimum hydrolytic activity towards PET; complete decomposition of PET to TPA and ethylene glycol | Optimum activity at 50 °C (close to PET Tg) | [16] |
| | *Pseudomonas* + *Bacillus* environmental consortium | Environmental enrichment | Synergistic PET degradation; complete conversion of BHET to TPA and ethylene glycol | Aerobic mesophilic incubation | [17] |
| PET, PCL, aromatic polyesters | *Thermobifida fusca*, *T. alba*, *T. cellulosilytica* | Compost (thermophilic actinomycetes) | Cutinases with broad substrate spectrum; aliphatic (PCL) and aromatic (PET, PTT) polyester hydrolysis | Thermophilic conditions (≥ 50 °C) | [18–21] |
| PBAT | Cutinases AaCut4 and AaCut10 | Marine fungus *Alternaria alternata* FB1 | High PBAT depolymerization efficiency; near-quantitative TPA recovery | Moderate temperatures (mild conditions) | [22] |
| | *Pseudomonas aeruginosa* S3, *Sphingobacterium* S2, *Geobacillus* EC-3 | Compost | Esterase secretion and PLA degradation under both mesophilic and thermophilic conditions | Mesophilic and thermophilic incubation | [23] |

| Polymer | Microorganism / Enzyme | Origin / Habitat | Degradation result | Experimental conditions | Ref. |
|---|---|---|---|---|---|
| PLA (UV-pretreated) | Dairy-sludge microbial consortium | Soil amended with dairy-industry sewage sludge | Complete degradation of PLA beverage cups | 15 days; thermophilic conditions; UV pretreatment of PLA | [24] |
| Mixed plastics (PE, PET, PS, LLDPE) | *Bacillus subtilis* RBM2 + *Fusarium oxysporum* RHM1; *Pseudomonas aeruginosa* RBM21 + *Bacillus subtilis* RBM2 | Composting of agricultural waste (ligninolytic microbiota) | Bacterial–fungal consortium increased PE degradation efficiency; bacterial–bacterial consortium showed broad-spectrum degradation of LLDPE, PET and PS | Aerobic, ligninolytic conditions | [25] |

## References


1. Khampratueng, P.; Rice, D.; Anal, A.K. Biodegradation of Low-Density Polyethylene by the Bacterial Strains Isolated from the Dumping Site Community. *Discov. Appl. Sci.* **2024**, *6*, doi:10.1007/s42452-024-06052-4.
2. Hussein, A.A.; Al-Mayaly, I.K.; Khudeir, S.H.; Hussein, A.A.; Al-Mayaly, I.K.; Kudier, S.H. Isolation, Screening and Identification of Low Density Polyethylene (LDPE) Degrading Bacteria from Contaminated Soil with Plastic Wastes Isolation, Screening and Identification of Low Density Polyethylene (LDPE) Degrading Bacteria from Contaminated Soil W. *Mesopotomia Environ. J.* **2015**, *1*, 1–14.
3. Ali, S.; Rehman, A.; Hussain, S.Z.; Bukhari, D.A. Characterization of Plastic Degrading Bacteria Isolated from Sewage Wastewater. *Saudi J. Biol. Sci.* **2023**, *30*, 103628, doi:10.1016/j.sjbs.2023.103628.
4. Esmaeili, A.; Pourbabaee, A.A.; Alikhani, H.A.; Shabani, F.; Esmaeili, E. Biodegradation of Low-Density Polyethylene (LDPE) by Mixed Culture of Lysinibacillus Xylanilyticus and Aspergillus Niger in Soil. *PLoS One* **2013**, *8*, doi:10.1371/journal.pone.0071720.
5. Ting, A.S.Y.; Sallahudin, A.Y. Biodegradation Potential of Mixed Cultures of Bacillus Sp. and Priestia Sp. from Landfill Investigated on Low-Density Polyethylene (LDPE) Sheets. *Water. Air. Soil Pollut.* **2025**, *236*, 1–17, doi:10.1007/s11270-025-08369-1.
6. Tao, X.; Ouyang, H.; Zhou, A.; Wang, D.; Matlock, H.; Morgan, J.S.; Ren, A.T.; Mu, D.; Pan, C.; Zhu, X.; et al. Polyethylene Degradation by a Rhodococcous Strain Isolated from Naturally Weathered Plastic Waste Enrichment. *Environ. Sci. Technol.* **2023**, *57*, 13901–13911, doi:10.1021/acs.est.3c03778.
7. Kong, D.; Zhang, H.; Yuan, Y.; Wu, J.; Liu, Z.; Chen, S.; Zhang, F.; Wang, L. Enhanced Biodegradation Activity toward Polyethylene by Fusion Protein of Anchor Peptide and Streptomyces Sp. Strain K30 Latex Clearing Protein. *Int. J. Biol. Macromol.* **2024**, *264*, 130378, doi:10.1016/j.ijbiomac.2024.130378.
8. Putcha, J.P.; Kitagawa, W. Polyethylene Biodegradation by an Artificial Bacterial Consortium: Rhodococcus as a Competitive Plastisphere Species. *Microbes Environ.* **2024**, *39*, 1–10, doi:10.1264/jsme2.ME24031.

9. Ibrahim, S.S.; Ionescu, D.; Grossart, H.P. Tapping into Fungal Potential: Biodegradation of Plastic and Rubber by Potent Fungi. *Sci. Total Environ.* **2024**, *934*, 173188, doi:10.1016/j.scitotenv.2024.173188.
10. Yu, Y.; Tian, K.; Hao, P.; Wang, Y.; Chang, M.; Zhou, D.; Gu, J.; Zhang, F.; Huo, H. Enzymatic Mechanism of Polystyrene Biodegradation: Key Depolymerization Enzymes in the Stenotrophomonas Maltophilia Sp. ZSL2493. *Environ. Res.* **2025**, *285*, 122659, doi:10.1016/J.ENVRES.2025.122659.
11. Parthasarathy, A.; Miranda, R.R.; Eddingsaas, N.C.; Chu, J.; Freezman, I.M.; Tyler, A.C.; Hudson, A.O. Polystyrene Degradation by Exiguobacterium Sp. RIT 594: Preliminary Evidence for a Pathway Containing an Atypical Oxygenase. *Microorganisms* **2022**, *10*, doi:10.3390/microorganisms10081619.
12. Zhang, T.; Li, X.; Rao, X.; Peng, Y.; Zhao, C.; Xu, Y.; Li, J.; Wei, J. Biodegradation of Polystyrene and Polyethylene by Microbacterium Esteraromaticum SW3 Isolated from Soil. *Ecotoxicol. Environ. Saf.* **2024**, *274*, 116207, doi:10.1016/j.ecoenv.2024.116207.
13. Yang, Y.; Yang, J.; Wu, W.M.; Zhao, J.; Song, Y.; Gao, L.; Yang, R.; Jiang, L. Biodegradation and Mineralization of Polystyrene by Plastic-Eating Mealworms: Part 1. Chemical and Physical Characterization and Isotopic Tests. *Environ. Sci. Technol.* **2015**, *49*, 12080–12086, doi:10.1021/acs.est.5b02661.
14. Yoshida, S.; Hiraga, K.; Takehana, T.; Taniguchi, I.; Yamaji, H.; Maeda, Y.; Toyohara, K.; Miyamoto, K.; Kimura, Y.; Oda, K. A Bacterium That Degrades and Assimilates Poly(Ethylene Terephthalate). *Science (80-. ).* **2016**, *351*, 1196–1199, doi:10.1126/science.aad6359.
15. Walter, A.; Sopracolle, L.; Mutschlechner, M.; Spruck, M.; Griesbeck, C. Biodegradation of Different PET Variants from Food Containers by Ideonella Sakaiensis. *Arch. Microbiol.* **2022**, *204*, 1–7, doi:10.1007/s00203-022-03306-w.
16. Sulaiman, S.; Yamato, S.; Kanaya, E.; Kim, J.J.; Koga, Y.; Takano, K.; Kanaya, S. Isolation of a Novel Cutinase Homolog with Polyethylene Terephthalate-Degrading Activity from Leaf-Branch Compost by Using a Metagenomic Approach. *Appl. Environ. Microbiol.* **2012**, *78*, 1556–1562, doi:10.1128/AEM.06725-11.
17. Roberts, C.; Edwards, S.; Vague, M.; León-Zayas, R.; Scheffer, H.; Chan, G.; Swartz, N.A.; Mellies, J.L. Environmental Consortium Containing Pseudomonas and Bacillus Species Synergistically Degrades Polyethylene Terephthalate Plastic. *mSphere* **2020**, *5*, 1–20, doi:10.1128/msphere.01151-20.
18. Müller, R.J.; Schrader, H.; Profe, J.; Dresler, K.; Deckwer, W.D. Enzymatic Degradation of Poly(Ethylene Terephthalate): Rapid Hydrolyse Using a Hydrolase from T. Fusca. *Macromol. Rapid Commun.* **2005**, *26*, 1400–1405, doi:10.1002/marc.200500410.
19. Acero, E.H.; Ribitsch, D.; Dellacher, A.; Zitzenbacher, S.; Marold, A.; Steinkellner, G.; Gruber, K.; Schwab, H.; Guebitz, G.M. Surface Engineering of a Cutinase from Thermobifida Cellulosilytica for Improved Polyester Hydrolysis. *Biotechnol. Bioeng.* **2013**, *110*, 2581–2590, doi:10.1002/bit.24930.
20. Wei, R.; Oeser, T.; Then, J.; Kühn, N.; Barth, M.; Schmidt, J.; Zimmermann, W. Functional Characterization and Structural Modeling of Synthetic Polyester-Degrading Hydrolases from Thermomonospora Curvata. *AMB Express* **2014**, *4*, 1–10, doi:10.1186/s13568-014-0044-9.
21. Then, J.; Wei, R.; Oeser, T.; Gerdts, A.; Schmidt, J.; Barth, M.; Zimmermann, W. A Disulfide Bridge in the Calcium Binding Site of a Polyester Hydrolase Increases Its Thermal Stability and Activity against Polyethylene Terephthalate. *FEBS Open Bio* **2016**, *6*, 425–432, doi:10.1002/2211-5463.12053.
22. Fei, F.; Su, Z.; Liu, R.; Gao, R.; Sun, C. Efficient Biodegradation of Poly(Butylene Adipate-Co-Terephthalate) in Mild Temperature by Cutinases Derived from a Marine Fungus. *J. Hazard. Mater.* **2024**, *480*, 136008, doi:https://doi.org/10.1016/j.jhazmat.2024.136008.
23. Noor, H.; Satti, S.M.; Din, S. ud; Farman, M.; Hasan, F.; Khan, S.; Badshah, M.; Shah, A.A. Insight on Esterase from Pseudomonas Aeruginosa Strain S3 That Depolymerize

Poly(Lactic Acid) (PLA) at Ambient Temperature. *Polym. Degrad. Stab.* **2020**, *174*, 109096, doi:10.1016/j.polymdegradstab.2020.109096.

24. Pattanasuttichonlakul, W.; Sombatsompop, N.; Prapagdee, B. Accelerating Biodegradation of PLA Using Microbial Consortium from Dairy Wastewater Sludge Combined with PLA-Degrading Bacterium. *Int. Biodeterior. Biodegrad.* **2018**, *132*, 74–83, doi:10.1016/j.ibiod.2018.05.014.

25. Salinas, J.; Martínez-Gallardo, M.R.; Jurado, M.M.; Suárez-Estrella, F.; López-González, J.A.; Estrella-González, M.J.; Toribio, A.J.; Carpena-Istán, V.; Barbani, N.; Cappello, M.; et al. Microbial Consortia for Multi-Plastic Waste Biodegradation: Selection and Validation. *Environ. Technol. Innov.* **2024**, *36*, doi:10.1016/j.eti.2024.103887.
